# Comparative metagenomic analysis reveals the adaptive evolutionary traits of siboglinid tubeworm symbionts

**DOI:** 10.3389/fmicb.2025.1533506

**Published:** 2025-04-17

**Authors:** Jinyi Liu, Yingli Zhou, Jingchun Feng, Chaofeng Cai, Si Zhang

**Affiliations:** ^1^Research Centre of Ecology and Environment for Coastal Area and Deep Sea, Guangdong University of Technology, Guangzhou, China; ^2^Southern Marine Science and Engineering Guangdong Laboratory (Guangzhou), Guangzhou, China; ^3^School of Ecology, Environment and Resources, Guangdong University of Technology, Guangzhou, China; ^4^Guangdong Basic Research Center of Excellence for Ecological Security and Green Development, Guangdong University of Technology, Guangzhou, China

**Keywords:** deep-sea environment, Siboglinid symbiont, comparative metagenome, adaptive evolutionary, metabolic diversity

## Abstract

Tubeworms flourish in marine cold seeps and hydrothermal vents through the establishment of symbiotic relationships with chemosynthetic bacteria. However, the environmental adaptations and evolutionary relationships of tubeworm symbionts across diverse habitats and hosts remain largely unknown. In this study, we characterized the genomes of 26 siboglinid tubeworm symbionts collected from deep-sea hydrothermal vents, cold seeps, and deep-sea mud, including two sequenced in this study and 24 previously published. Phylogenetic analysis classified the 26 symbiont genomes into five distinct clusters at the genus level. The findings highlight the remarkable diversity in symbiont classification, influenced by the habitat and species of tubeworm, with the symbiont genome characteristics of various genera revealing unique evolutionary strategies. Siboglinid symbionts exhibit functional metabolic diversity, encompassing chemical autotrophic capabilities for carbon, nitrogen, and sulfur metabolism, hydrogen oxidation, and a chemoorganotrophic ability to utilize various amino acids, cofactors, and vitamins. Furthermore, the symbiont’s homeostatic mechanisms and CRISPR-Cas system are vital adaptations for survival. Overall, this study highlights the metabolic traits of siboglinid symbionts across different genera and enhances our understanding of how different habitats and hosts influence symbiont evolution, offering valuable insights into the strategies that symbionts use to adapt and thrive in extreme environments.

## 1 Introduction

Deep-sea organisms exhibit a multitude of adaptive mechanisms due to the harsh environmental conditions they face, such as extreme pressure, darkness, low oxygen levels, and the presence of toxic substances like sulfides and heavy metals, along with limited food availability (Ramirez-Llodra et al., 2010). Notably, the unpredictable nutrient supply compels deep-sea invertebrates to form symbiotic partnerships with chemosynthetic microbes to secure essential nutrients (Dubilier et al., 2008; Husnik et al., 2021). To maintain efficient chemosynthesis, the host animal must engage with the chemosynthetic symbiont at the tissue, cellular, and molecular levels (Hinzke et al., 2019). The host supplies essential gasses, such as hydrogen sulfide, methane, oxygen, and carbon dioxide, thereby promoting rapid symbiont growth (Hinzke et al., 2021). Moreover, hosts regulate the symbiont population size by selectively digesting a portion of it and extracting and transporting nutrients to support their growth (Gómez-Mendikute et al., 2005; Hirokawa and Noda, 2008; Renoz et al., 2015). These unique symbiotic relationships between deep-sea invertebrates and chemosynthetic bacteria serve as valuable models for studying animal–microbe interactions and have been widely researched.

Deep-sea tubeworms, as members of the siboglinid group, are invertebrates lacking mouths and digestive tracts as adults (Bright et al., 2013). Four lineages of tubeworms have been identified: Vestimentifera, the genus *Sclerolinum*, the clade Frenulata, and the genus *Osedax* (Li Y. et al., 2015; Li et al., 2017a). Vestimentifera tubeworms are split into two main groups (Bright and Lallier, 2010): one is found solely in the Pacific Ocean’s hydrothermal vents, including *Riftia*, *Oasisia*, *Tevnia*, and *Ridgeia*; and the second group primarily consists of *Lamellibrachia*, *Escarpia*, *Paraescarpia,* and *Seepiophila*, which are found in cold seeps worldwide. Additionally, *Escarpia spicata*, *Lamellibrachia barhami*, *Lamellibrachia satsuma*, and *Lamellibrachia anaximandri* are found in hydrothermal vents and cold seeps (Bright and Lallier, 2010). These organisms mainly form symbiotic relationships with sulfur-oxidizing bacteria to support growth (Bright et al., 2013). The symbiotic bacteria reside within specialized host cells in a highly vascularized internal organ called trophosome (Hand, 1987). Deep-sea tubeworms acquire endosymbiotic bacteria through horizontal transmission, with each generation obtaining these symbionts from the environment (Nussbaumer et al., 2006; Harmer Tara et al., 2008). Previously, it was hypothesized that Vestimentifera tubeworms harbored a single endosymbiont phylotype (γ-proteobacteria) (Bright and Lallier, 2010). However, recent studies have identified two distinct γ-proteobacteria phylotypes in *L. anaximandri* tubeworms (Zimmermann et al., 2014), while both γ-proteobacteria and *ε*-proteobacteria have been detected in *L. satsuma* (Patra et al., 2016).

Tubeworms absorb hydrogen sulfide and oxygen from their surroundings, using hemoglobin to transport sulfide to sulfur-oxidizing bacteria (Dubilier et al., 2008). This sulfur oxidation process transforms toxic hydrogen sulfide into monosulfur or thiosulfate, thereby reducing the concentration of hydrogen sulfide within the host. The chemical energy released from this oxidation is used in two carbon dioxide fixation pathways (Li et al., 2018): the Calvin–Benson–Bassham cycle (CBB) and the reductive tricarboxylic acid cycle (rTCA). These pathways generate sugars that serve as a carbon source for tubeworm growth and metabolism (Bright and Giere, 2005). Previous research has demonstrated that habitat type influences the diversification of chemosynthetic symbionts, and the mechanism of interaction between symbionts and their hosts has been thoroughly investigated (Dubilier et al., 2008; Perez and Juniper, 2016; Hinzke et al., 2019). The host provides the basic conditions and environment necessary for the survival of the symbionts, such as nutritional sources, and influences the physiological traits and adaptability of the symbionts (Hinzke et al., 2021). In contrast, the habitat determines the changes in environmental conditions, such as temperature, pressure, and chemical composition, which are crucial for the symbionts’ adaptability (Childress and Girguis, 2011). However, the variations in ecological functions and the evolutionary relationships of symbiotic bacteria across different habitats and tubeworm hosts remain unclear. Considering that various siboglinid tubeworms inhabit a wide range of chemosynthetic habitats, from hydrothermal vents and cold seeps to deep-sea muddy environments, it is crucial to analyze the diversity, metabolism, organic deposits and evolutionary relationships of symbionts across these habitats and in different tubeworm hosts to uncover the adaptive mechanisms of siboglinid symbionts.

The present study sequenced the symbiont genomes of the large tubeworm *Paraescarpia echinospica* and the fine tubeworm *Sclerolinum* sp., both of which were collected from the Haima cold seeps in the South China Sea. Additionally, a total of 26 tubeworm symbionts collected from various global locations, including the two symbionts analyzed in this study, were classified into five genera for comparative analysis: *Sulfurovum*, SZUA-229, *Candidatus* Vondammii, *Candidatus* Endoriftia, and QGON01. This is the first study to analyze the characteristics of siboglinid symbionts at the genus level. In this study, we aim to investigate the commonalities and heterogeneity among symbionts from five different genera. By comprehensively analyzing their phylogenetic relationships, genomic features, functional metabolic diversity, nutrient synthesis capabilities, and homeostatic mechanisms, we seek to elucidate the adaptation strategies employed by these symbionts in extreme environments. The results showed that the genomes of the five genera exhibit significant differences, with *Candidatus* Vondammii symbionts being more sensitive to environmental changes. Sulfur metabolism functional genes are highly conserved across these genera. The *Candidatus* Vondammii symbionts possess an additional NAD(P) (+)-reducing hydrogenase pathway, which may contribute to their metabolic flexibility. It is emphasized that siboglinid symbionts synthesize a variety of amino acids to meet their nutritional needs. Terpene compounds seem to facilitate the adaptation of QGON01 symbionts to the host’s internal environment. Furthermore, CRISPR-Cas proteins in siboglinid symbionts provide a defense mechanism against foreign bacteriophages and viral invasions. Our findings will enhance our understanding of the ecological roles played by deep-sea symbiotic microorganisms.

## 2 Materials and methods

### 2.1 Sample collection

Specimens of *P. echinospica* and *Sclerolinum* sp. tubeworms were sourced from the Haima cold seeps in the South China Sea (Since we could not determine the *Sclerolinum* species, we consistently apply “*Sclerolinum* sp.” in this study.). The collection of *P. echinospica* was carried out in April 2023 from a site of 1,385 meters depth (110°27′5,310″E, 16°43′5,017″N). *Sclerolinum* sp. samples were collected in July 2022 from a depth of 1,428 meters. The Remotely Operated Vehicle (ROV) is powered by the mother ship through a cable, allowing operators aboard the mother ship to remotely control the ROV and collect tubeworms. The tubeworms were placed into an insulated “bio-box” with a sealed lid to minimize temperature fluctuations of the water within the container. Upon boarding the vessel, they were immediately stored in a −80°C freezer.

### 2.2 DNA extraction, metagenomic library, and sequencing

A single specimen each of *P. echinospica* and *Sclerolinum* sp. was processed for metagenomic DNA extraction using (Supplementary Figure S1). The *P. echinospica* was dissected using sterile instruments, with the plume (a gill-like structure), vestimentum (mainly composed of muscle), and trophosome (an organ housing symbionts) (Supplementary Figure S1). Since the symbiotic bacteria filled the entire trophosome (Nussbaumer et al., 2006), we selected trophosome tissue of approximately 1 cm^3^ in size for the extraction experiment. Additionally, because the tubes of *Sclerolinum* sp. are narrow and soft, making direct dissection difficult, we minced the tubes, along with the trophosome tissue, directly for the extraction experiment (Supplementary Figure S1). The samples were thoroughly rinsed with DNA extraction buffer (100 mM Tris–HCl, 100 mM EDTA, 100 mM Na_2_HPO_4_, 1.5 M NaCl, 1% CTAB, pH 8.0) to eliminate surface impurities and preservatives. The trophosome tissue was immersed in 1 mL of the same buffer, minced with sterile scissors, and incubated at room temperature for 20 min. After the larger tissue fragments had settled, the supernatant containing microbial cells was separated. High-speed centrifugation (12,000 rpm, 13,201 g) was performed at room temperature for 3 min to pellet the microbes for further analysis.

A magnetic bead-based soil and fecal genomic DNA extraction kit (TIANGEN) was used for DNA extraction and purification as per the manufacturer’s guidelines. The purified DNA was then subjected to quantitative and qualitative analysis using a Qubit 4.0 fluorimeter and 1% agarose gel electrophoresis, respectively (Lee et al., 2012). The VAHTS Universal Plus DNA Library Prep Kit for Illumina was employed to prepare metagenomic libraries according to the manufacturer’s instructions. Finally, the metagenomic libraries were sequenced on the Illumina Novaseq 6000 platform using the PE150 strategy at Shanghai Personalbio Bioscience and Technology (Shanghai, China) with 10 Gb for each library.

### 2.3 Metagenome assembly and functional annotation

The raw sequencing data were quality controlled using Fastp v0.23.2 (Chen, 2023) (parameters: -q 20 -u 20 -l 50 -w 40–5 -M 30 -g -D -dup calc accuracy 6) to remove adapters and low-quality reads. The clean reads obtained from each sample were individually assembled using MEGAHIT v1.2.9 (Li D. et al., 2015; Li et al., 2016) (parameters: -t 20 -m 0.96 --no-mercy --kmin-1pass) with k-mer sizes of 21, 29, 39, 49, 59, 69, 79, 89, 99, 99, 109, 119, 129, and 141 bp. The assembled metagenome contigs (≥2,000 bp) were binned using the binning module within MetaWRAP v1.3.2 (parameters: -m 500 -t 40, −c 50 -x 10 –quick), which also selected the best representative genomes through its Bin refinement module (Uritskiy et al., 2018). The quality of the metagenome-assembled genomes (MAGs) was assessed using Quast v5.2.0 (Mikheenko et al., 2018) and checkM2 (Parks et al., 2015) (parameters: --allmodels -x fasta --force -t 8). Prodigal v2.6.3 (Hyatt et al., 2012) (parameters: -o .fna -a .faa -p meta) was employed to predict the proteins and genes for all MAGs. The protein sequences were functionally annotated using the Kyoto Encyclopedia of Genes and Genomes (KEGG) database through KofamScan (v.1.1.0) (Aramaki et al., 2019). Additionally, functional classifications were assigned based on Clusters of Orthologous Groups using eggNOG-mapper v2 (Cantalapiedra et al., 2021).

### 2.4 Genomic comparisons and phylogenetic analyses of the siboglinid symbionts

**Figure 1:**
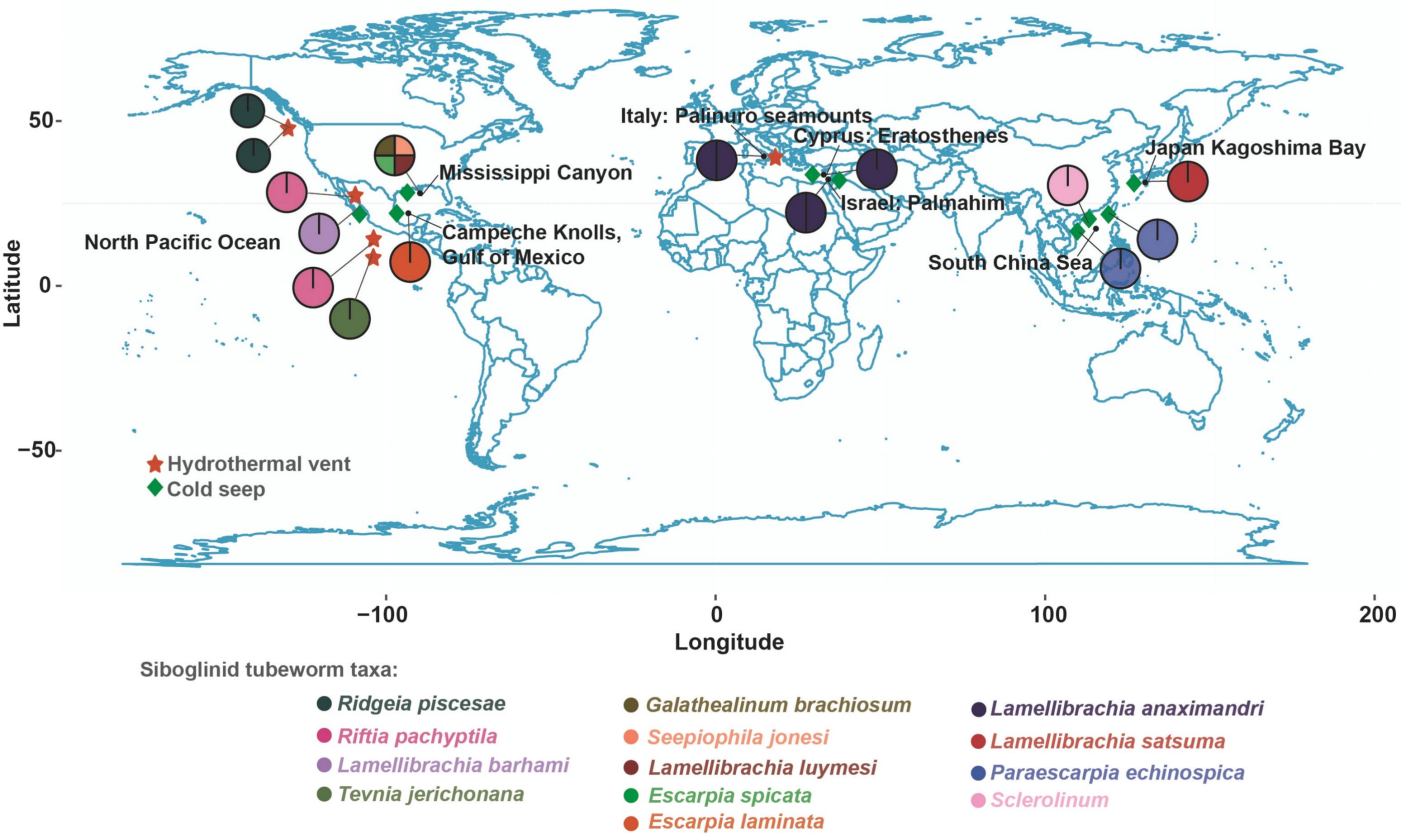
Geographical distribution of siboglinid tubeworms. The black font in the figure represents locations, while the circles represent tubeworms. The colors of the circles correspond to the text in the “Siboglinid tubeworm taxa” section below.

The genomes of 24 tubeworm symbionts (Figure 1; Supplementary Table S1) downloaded from the National Center for Biotechnology Information (NCBI) database were analyzed and compared with the two symbiont genomes recovered in this study. We used GTDB-TK v2.2.6 with the default settings to perform taxonomic classification for the MAGs (Parks et al., 2018; Chaumeil et al., 2019). The ANIb model in PyAni v0.2.10 (parameters: -m ANIb -g) was used to calculate average nucleotide identity (ANI) values for all genome pairs (Goris et al., 2007; Richter and Rosselló-Móra, 2009). The pheatmap package in R was employed to generate heatmaps. After KEGG annotation, Venn diagrams were created using the Venn tool in the Origin and UpSetR packages to represent shared and unique genes between symbionts. Phylogenetic trees were constructed based on 112 conserved proteins extracted using GTDB-TK and automatically selected best model of IQ-TREE v2.2.6 and branch support estimated using 1,000 ultrafast bootstrap replicates (Minh et al., 2020). The iTOL editor was utilized to visualize the evolutionary trees (Letunic and Bork, 2021). Homologous gene cluster identification of symbionts was performed using Orthofinder (parameters: -t 24 -a 8 -M msa -S blast -A mafft -T iqtree) (Emms and Kelly, 2019).

### 2.5 Pangenomic analysis

Pangenome analysis was carried out using the PPanGGOLiN tool^1^ to characterize the pangenome across 26 tubeworm symbiont genomes. PPanGGOLiN partitions the nodes using an Expectation–Maximization algorithm based on a multivariate Bernoulli mixture model coupled with a Markov Random Field (Bazin et al., 2020; Gautreau et al., 2020). This approach considers the graph’s topology and the presence or absence of genes in pangenomes to classify tubeworm symbiont gene families into persistent, cloud, and one or several shell partitions (Gautreau et al., 2020). Persistent genomes contain gene families found in most genomes; shell genomes have them at moderate frequencies, and cloud genomes have them at low frequencies. Specific definitions of the three partitions can be found in the Supplementary material.

## 3 Results and discussion

### 3.1 Symbiont genomes in deep-sea tubeworms

**Figure 2:**
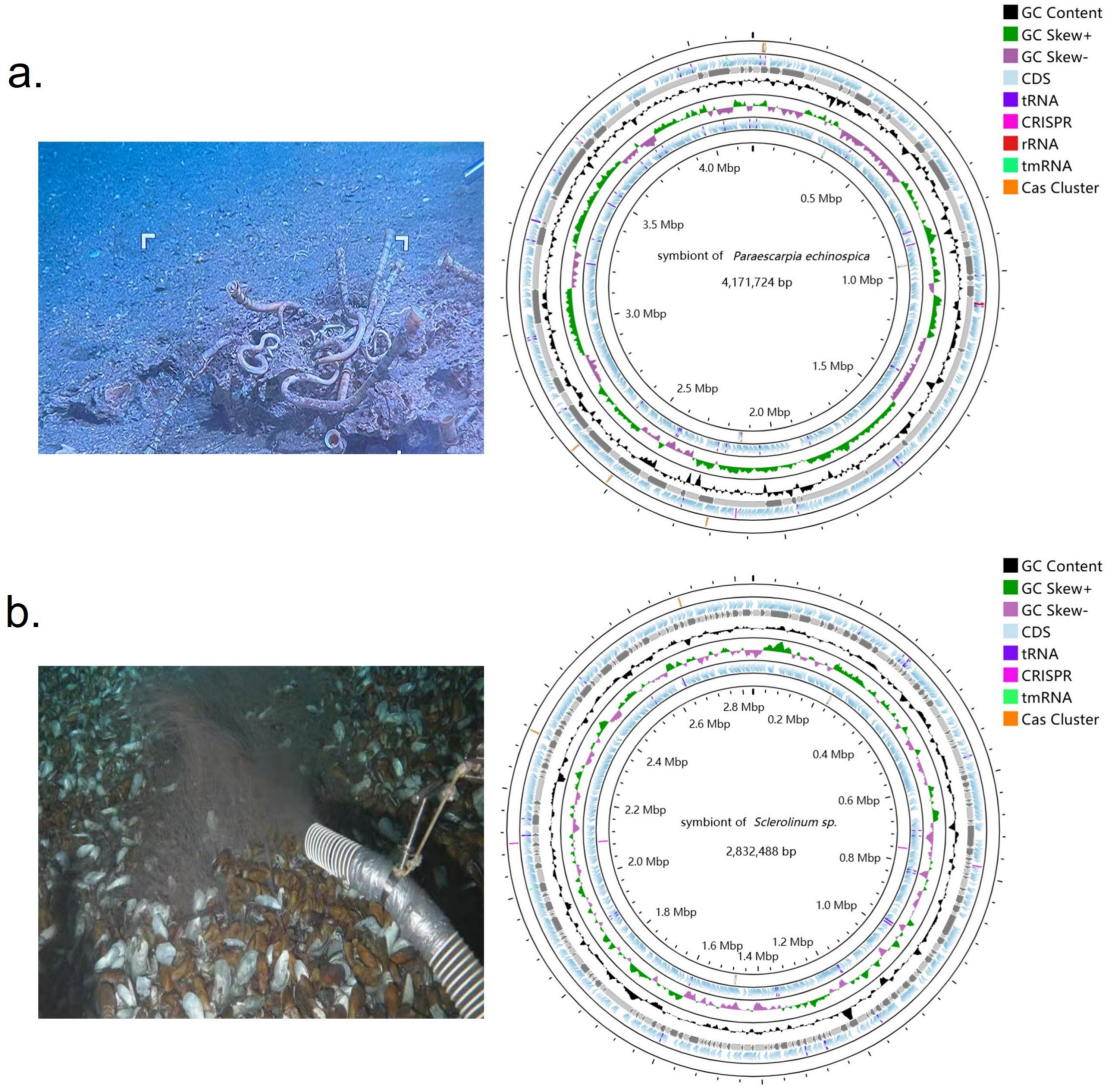
Tubeworm sampling and symbiont genome circle map. **(a)** Sampling map of *Paraescarpia echinospica* and key features of the symbiont genome. From the innermost to the outermost, the first circle displays Cas Cluster and CRISPR sequences, the second circle shows the GC skew, the third circle represents the GC content, the fourth circle illustrates open reading frames (CDS, tRNA, rRNA, and tmRNA), and the fifth circle indicates the Cas Cluster. **(b)** Sampling map of *Sclerolinum* sp. and key features of the symbiont genome. From the innermost to the outermost, the first and fifth circles display the Cas Cluster and CRISPR sequences, the second circle shows the GC skew, the third circle represents the GC content, and the fourth circle illustrates open reading frames (CDS, tRNA, and tmRNA). The genomic features were plotted using the CGView Server (http://stothard.afns.ualberta.ca/cgview_server/). CDS, coding sequence.

Tubeworm symbionts, *P. echinospica* (Hai6C03) and *Sclerolinum* sp. (SCTW1H), were collected from the Haima cold seeps in the South China Sea to construct a metagenome. Genome quality assessment using CheckM2 indicated that the Hai6C03 symbiont genome assembly exhibited the highest completeness and least contamination compared to other *P. echinospica* symbiont genomes (Supplementary Table S2). SCTW1H also exhibited the lowest level of contamination compared to similar genomes. The assembled Hai6C03 genome (Figure 2a) had a size of 4,171,724 bp, a GC content of 54%, a completeness of 99.33%, and a contamination of 0.3%. The assembled SCTW1H genome (Figure 2b) had a size of 2,832,488 bp, a GC content of 50%, a completeness of 98.38%, and a contamination of 0.48%.

All tubeworm symbiont genomes were collected from public databases as of July 2023 to analyze the heterogeneity among deep-sea tubeworm symbiont genomes. The ANI results indicate that Vestimentifera tubeworms inhabiting hydrothermal vents in the Pacific Ocean have symbionts that are highly similar to one another. In contrast, Vestimentifera tubeworms from cold seeps exhibit greater variation in their symbiont similarities (see Supplementary Figure S2). Notably, the *L. anaximandri* tubeworms can live in both hydrothermal and cold seep environments and host different species of symbionts. The ANI results revealed that the symbionts of *L. anaximandri* found in hydrothermal vents (GCA_016756855 and GCA_016756895) were similar to those in cold seeps (GCA_016756955), with a similarity exceeding 0.98, suggesting they belong to the same species. Additionally, the GCA_016756865 and GCA_016756835 symbionts from the cold seep showed a similarity of 0.999, indicating they also belong to the same species. However, the similarity between the GCA_016756955 and GCA_016756865 symbionts from the cold seep was only 0.93, suggesting they are distinct types of symbionts. Considering the ANI diversity among *L. anaximandri* symbionts, we speculate that adaptive evolution occurs as these symbionts colonize the tubeworms, enabling them to better adapt to their host environments. More detailed ANI information is available in the Supplementary material.

**Figure 3:**
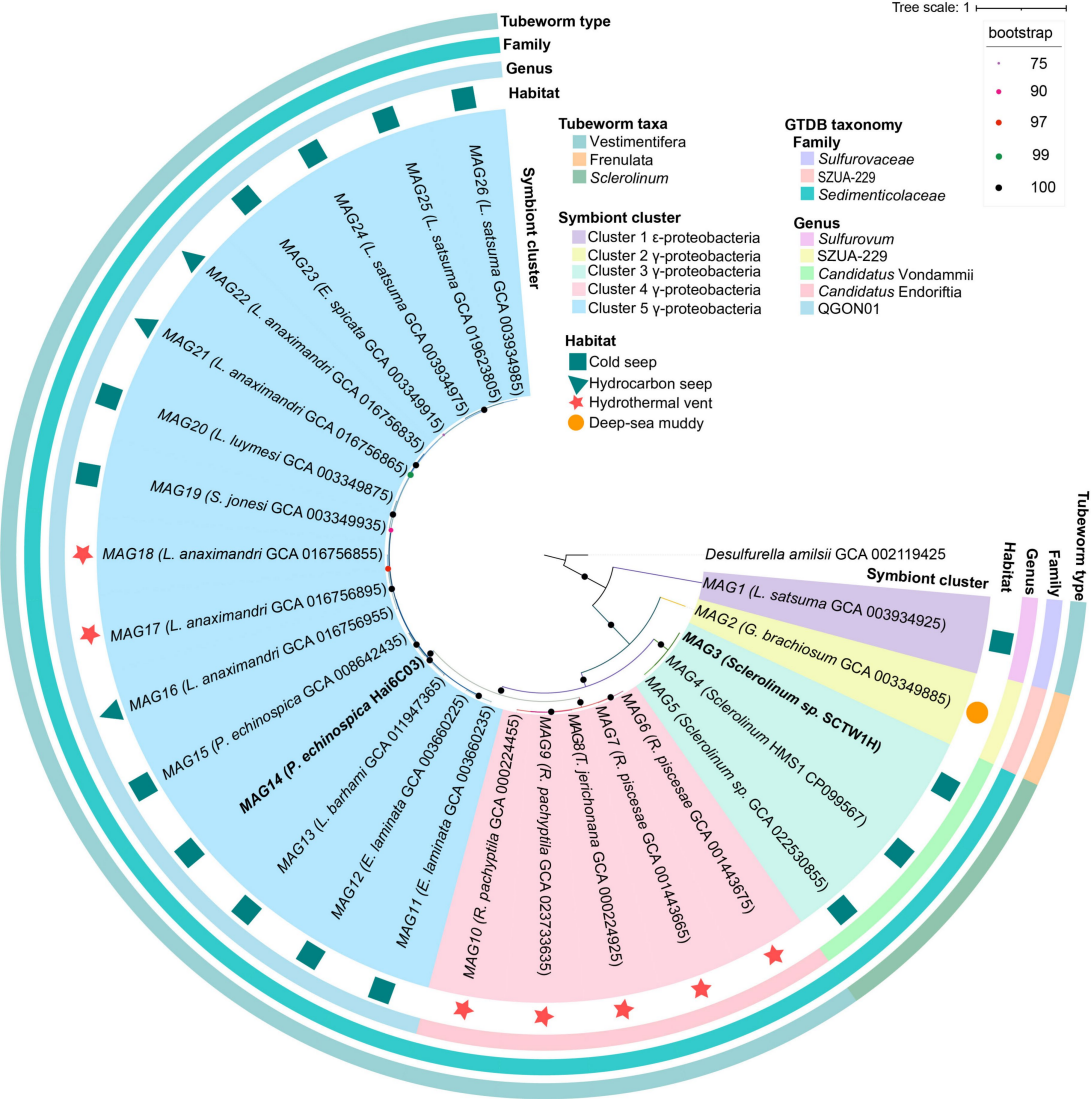
Phylogenetic tree of 26 tubeworm symbionts. Conserved protein sequences were sourced from GTDB-TK. The maximum likelihood tree was constructed using IQtree, incorporating 1,000 replicates and the best-fit model automatically selected by ModelFinder. Node circles represent ultrafast bootstrap support values (percentages), with the color scale indicated in the upper-right legend panel. *Desulfurella amilsii* was used as an outgroup. The two bolded symbionts were contributed specifically for this study. MAG stands for metagenome-assembled genome, with the number representing the sequence number. The contents of the parentheses indicate the host type and the genome number of the symbiont.

To examine the differences in ANI similarity and the evolutionary relationships of deep-sea tubeworm symbionts, this study constructed a phylogenetic tree of siboglinid symbionts, which formed five branches (Figure 3). In line with previous research (Yang et al., 2020), siboglinid symbionts within the Vestimentifera lineage exhibited habitat-related patterns, forming two distinct clades: one associated with hydrothermal vents and one associated with cold seeps. Notably, regardless of whether the *L. anaximandri* symbionts are found in hydrothermal or cold seep environments, they belong to the cold seep clade (Figure 3). This result suggests that for this class of tubeworms, which can inhabit hydrothermal vents and cold seeps, the relatives remain similar and are part of the same evolutionary lineage despite the presence of two species of symbionts.

The results of GTDB-TK classification aligned with the phylogenetic tree, dividing siboglinid symbionts into five groups at the genus level (Supplementary Table S3; Figure 3). The classification of siboglinid symbionts revealed significant diversity based on host species and habitat characteristics. The 26 siboglinid symbionts are distributed across three families: *Sedimenticolaceae*, *Sulfurovaceae*, and SZUA-299, with *Sulfurovaceae* being the second symbiont type found in *L. satsuma*. These symbionts are further classified into five genera: QGON01 (16 symbionts), *Candidatus* Endoriftia (five symbionts), *Candidatus* Vondammii (three symbionts), *Sulfurovum* (one symbiont), and SZUA-229 (one symbiont). The names QGON01 and SZUA-229 are provisional genus-level clusters derived from the GTDB genomic classifications (Parks et al., 2022). QGON01, *Candidatus* Endoriftia, and *Sulfurovum* are members of the Vestimentifera lineage, while *Candidatus* Vondammii is classified within the *Sclerolinum* clade, and SZUA-299 falls under the Frenulata group. The symbionts associated with the tubeworm *L. anaximandri* from both hydrothermal vent and cold seep environments belong to the QGON01 genus (Figure 3). In conclusion, the analysis suggests that the similarity among siboglinid symbionts is primarily driven by their habitat and host species. The findings suggest that host species exert a greater influence on symbiont evolution than habitat, highlighting the importance of host selection for symbiont survival.

### 3.2 Genomic features of symbionts

**Figure 4:**
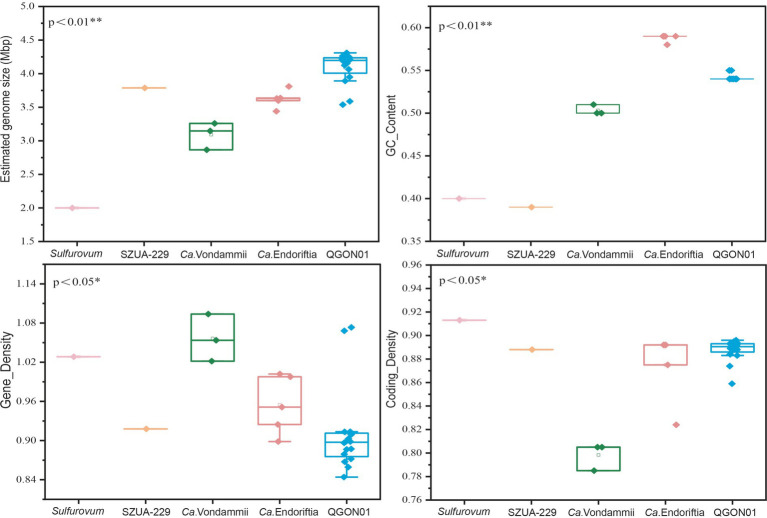
Genomic characterization of siboglinid symbionts at the genus level. **(A)** Estimated size of the symbiont genome. **(B)** GC content of the symbiont genome. **(C)** Gene density of the symbiont genome, represented by the number of genes per 1,000 bp. **(D)** Coding gene density in the symbiont genome. The analysis was conducted using the Kruskal–Wallis test statistic. *p*-values less than 0.05 or 0.01 indicate significant differences, with * denoting *p* < 0.05, and ** denoting *p* < 0.01. *Candidatus* Vondammii (*Ca.* Vondammii), *Candidatus* Endoriftia (*Ca.* Endoriftia).

To investigate the influence of varying environments and host selections on symbiont genomes, we assessed whether the four key metrics (genome size, GC content, gene density, and coding density) differed significantly among the five genera (Figure 4). We used the Kruskal-Wallis test to calculate the significance levels for genome size, GC content, gene density, and coding density of each genus (Theodorsson-Norheim, 1986). Notably, QGON01 symbionts from cold seeps had a significantly larger average genome size than *Candidatus* Endoriftia symbionts collected from hydrothermal vents (Kruskal-Wallis test, *p* < 0.01) (Figure 4A). Additionally, the GC content of Vestimentifera tubeworm symbiont genomes displayed an environment-dependent pattern. *Candidatus* Endoriftia symbionts collected from hydrothermal vents exhibited a significantly higher GC content compared to other genera (Kruskal-Wallis test, *p* < 0.01) (Figure 4B). The relationship between genome size and gene density is inverse in siboglinid symbionts, with larger genomes exhibiting smaller gene densities (Kruskal-Wallis test, *p* < 0.05) (Figure 4C). Widely distributed bacteria have large genomes that help them adapt to various environments (Zhou et al., 2022). The Vestimentifera tubeworms are found in cold seeps globally (Figure 1), which has led to the QGON01 symbiont exhibiting a large genome. Additionally, genome size is correlated with nutrient limitation (Giovannoni et al., 2014), which suggests that symbionts residing in nutrient-limited environments gain survival advantages by minimizing metabolic costs. Generally, the smallest and most compact genomes exhibit higher gene density due to a reduced allocation of genomic space to non-coding DNA (Giovannoni et al., 2005; Bobay and Ochman, 2017). Adaptation to extreme environments is a significant factor influencing variations in GC content. High GC content in *Candidatus* Endoriftia symbionts indicates their adaptation to varying environmental conditions caused by thermal gradients and oxidative stress (Teng et al., 2023), leading to higher energy consumption and enhanced thermal stability. In contrast, symbionts from other genera exhibit lower GC content, a consequence of their adaptation to cold seep environments (Teng et al., 2023). The growth rate of tubeworms in unstable hydrothermal vent environments is much higher than that of those in cold seep environments (Bright et al., 2013). This difference suggests that symbionts in hydrothermal vent environments must have a faster metabolic rate to provide sufficient nutrients to their hosts. The *Candidatus* Vondammii symbiont genome was found to have the highest gene density but the lowest coding density (Kruskal-Wallis, *p* < 0.05) (Figure 4D). This is due to the loss of many genes in the *Candidatus* Vondammii symbionts, leading to a simplified metabolic capacity (Gao et al., 2023).

We used the PPanGGOLiN software to conduct a pangenomic analysis and identify trends in the gene families of tubeworm symbionts related to persistent, shell structure, and cloud formation (Bazin et al., 2020; Gautreau et al., 2020). Heatmaps illustrating the distribution of gene families among tubeworm symbionts in the persistent, cloud, and shell categories reveal significant differences across various genera (see Supplementary Figure S4). Notably, the gene family distribution of *L. anaximandri* symbionts found in hydrothermal vents is consistent with that of *L. anaximandri* symbionts in cold seeps. This finding suggests that the tubeworm host has a more pronounced effect on the distribution of symbiont gene families than the habitat itself.

We predict evolutionary trends in siboglinid symbiont gene family partitioning by rarefaction curves (Supplementary Figures S5, S6). The “persistent” curve of the siboglinid symbionts plateaued, indicating that genes essential for key metabolic pathways and biosynthetic capabilities remained largely constant in the symbiont genome. The “shell” curve changed minimally, suggesting that genes acquired through horizontal gene transfer, such as those encoding functions related to environmental adaptation, pathogenicity, virulence, or secondary metabolites, exhibited minor variations. The “cloud” curve showed an upward trend, implying that genes acquired through horizontal gene transfer, such as phage-related genes, antibiotic resistance genes, and plasmid genes, increased continuously. We speculate that the trend of core metabolic genes is stable in siboglinid symbionts, indicating that the symbionts can steadily produce energy to meet the nutritional requirements of both themselves and their hosts. At the same time, the related secondary metabolites showed small fluctuations. The increasing trend of phage and other related genes suggests that phages have complex interactions with the tubeworm symbionts. Specifically, phages play an important role in the evolution of tubeworm symbionts by influencing their metabolism, environmental adaptation, and facilitating nutrient release (Han et al., 2024; Huang et al., 2024). For example, genes involved in carbon metabolism (*korABCD*, *sucDC*, *sdhB*, *mdh*, *por*, *rbcL*, *PGK*, *GAPDH*, *TKT*, and *PRK*), nitrogen metabolism (*napAB*, *norBC*), and sulfur metabolism (*aprAB*, *soxYZB*, and *dsrAB*) are primarily located in the persistent partition. Additionally, genes related to the biosynthesis of valine, leucine, and isoleucine are also present in the persistent partition. In the shell partition, genes involved in pantothenate and CoA biosynthesis, biotin metabolism, and terpenoid backbone biosynthesis are predominantly distributed. In the cloud partition, genes related to prokaryotic defense systems, the biosynthesis of ansamycins, the biosynthesis of enediyne antibiotics, carbapenem biosynthesis, and streptomycin biosynthesis are found. A more detailed distribution of these genes is provided in the Supplementary Table S4.

### 3.3 Functional characterization of symbionts

#### 3.3.1 Functional gene classification of symbionts

**Figure 5:**
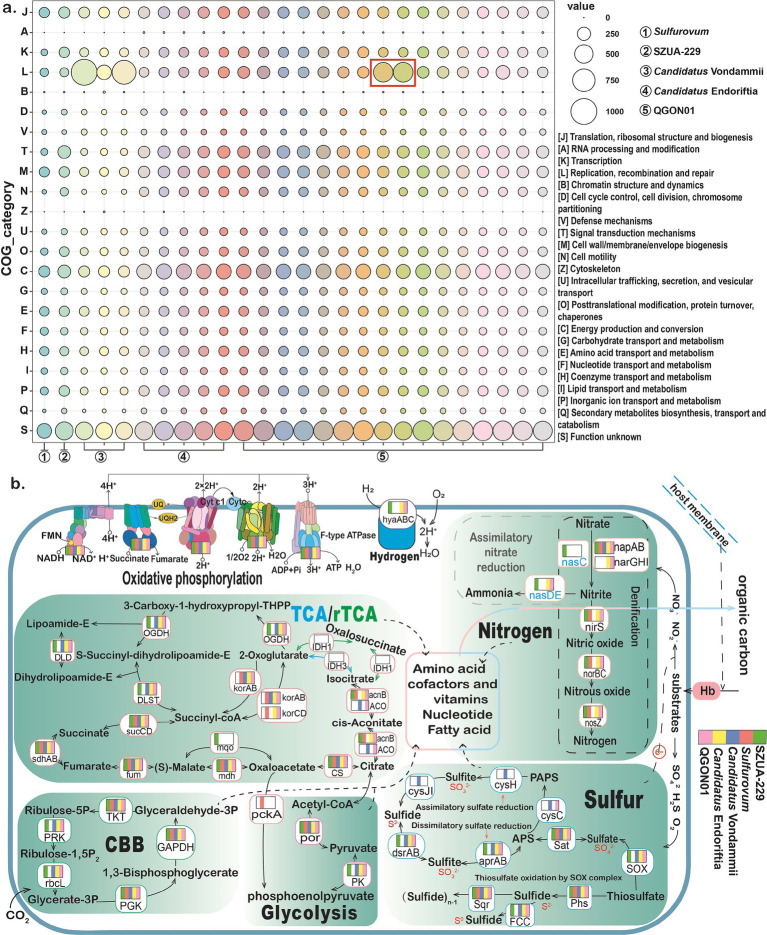
Functional analyses of siboglinid symbionts. **(a)** Map illustrating the number of genes functionally categorized and annotated by eggNOG-mapper protein sequences. Circled red boxes highlight the symbiont genomes of *S. jonesi* and *L. luymesi*. The circle colors are used for visual distinction and do not reflect specific classifications. **(b)** Map of the central metabolic pathways in symbionts from different genera. The boxes and colors on the genes represent the presence or absence of the gene in the symbionts of the five genera. Green represents the genus SZUA-229, red represents *Sulfurovum*, blue represents *Candidatus* Vondammii, yellow represents *Candidatus* Endoriftia, and pink represents QGON01. The presence of the corresponding color on a gene indicates its presence in the symbionts of that genus. Note: the absence of detectable genes does not rule out their existence due to potential genome incompleteness.

To investigate the adaptive differences of symbionts across various environments and hosts, this study examined their functional characteristics. Our statistical analysis of the number of functional genes in symbionts from different habitats shows that those from hydrothermal vents and hydrocarbon seep environments exhibit greater enrichment of genes involved in carbon metabolism, nitrogen metabolism, and sulfur oxidation. In contrast, symbionts from cold seeps show the greatest variation in gene enrichment related to carbon and sulfur metabolism, ranging from the highest to the lowest levels (Supplementary Figure S7). This result supports our hypothesis that symbionts from hydrothermal vents exhibit faster energy metabolism rates. The distribution of tubeworms in cold seeps worldwide suggests that differences in functional metabolic gene enrichment in symbionts are driven by adaptations to distinct environmental conditions. Additionally, we analyzed the number of functional genes in the symbionts from three lineages: Vestimentifera, Frenulata, and *Sclerolinum*. The symbionts of Vestimentifera showed the highest enrichment of genes involved in carbon metabolism, nitrogen metabolism, and sulfur oxidation, followed by those of Frenulata, which showed lower enrichment (Supplementary Figure S8). In contrast, the symbionts of *Sclerolinum* exhibited the lowest gene enrichment in these metabolic pathways (Supplementary Figure S8). However, *Sclerolinum* symbionts (*Candidatus* Vondammii) had a higher number of genes related to replication, recombination, and repair compared to those of the other lineages (Figure 5a). These findings suggest that the nutritional constraints of the host influence the symbionts’ functional gene composition. This is because, compared to *Sclerolinum* tubeworms, Vestimentifera and Frenulata tubeworms possess thicker and larger tubes, with their trophosome comprising nearly half of their body volume (Figure 2a). This suggests that Vestimentifera and Frenulata tubeworms can accommodate a greater abundance of sulfur-oxidizing bacteria and provide more substrates for these symbionts, enabling them to utilize more sulfide for energy metabolism (Nussbaumer et al., 2006; Li et al., 2018). Conversely, *Sclerolinum* sp. tubeworms have thin, soft tubes, with the red trophosome visible through the tube (Supplementary Figure S1). This suggests that *Candidatus* Vondammii symbionts require more genes involved in DNA replication, recombination, and repair functions to ensure efficient genome replication, growth, and reproduction in resource-limited environments (Yue et al., 2022). Additionally, the symbiont’s cellular motility aids in concentrating the symbiont in an optimal location for growth within the host and enhances its ability to perceive its environment and respond to external stimuli (Aschtgen et al., 2019; Li et al., 2020). Signal transduction mechanisms play a significant regulatory role in symbionts, assisting them in adapting and responding to environmental changes (Laub and Goulian, 2007). Consistent with the findings of Gao et al. (2023) *Candidatus* Vondammii symbionts lack gene sets encoding environmental sensing and motility (Figure 5a). Therefore, we propose that *Sclerolinum* symbionts are more sensitive to environmental changes than symbionts of any other genus. In conclusion, siboglinid symbionts exhibit a unique mutualistic strategy with their host to adapt to environmental changes.

#### 3.3.2 Energy sources

##### 3.3.2.1 Carbon fixation

Previous research has demonstrated that the tubeworm sulfur autotrophic symbiont employs the CBB pathway and the rTCA cycle for carbon fixation (Markert et al., 2007; Robidart et al., 2008; Gardebrecht et al., 2012). The present findings suggest that, excluding the *Sulfurovum* symbiont, all siboglinid symbionts possess the core components of the CBB cycle (Figure 5b). This includes ribose phosphate diketone carboxylase oxidase (*rbcL*), the most abundant enzyme in the biosphere, which is capable of fixing approximately 90% of the inorganic carbon (Erb and Zarzycki, 2018). Within the rTCA cycle, isocitrate dehydrogenase (*IDH*) is absent in all siboglinid symbionts, while 2-methylisocitrate dehydratase (*acnB*) is missing in the *Candidatus* Vondammii symbiont. However, *acnB* was identified in eight genomes, including one *Sulfurovum* symbiont, one SZUA-229 symbiont, three *Candidatus* Endoriftia symbionts (found in *R. pachyptila* and *T. jerichonana*), and three QGON01 symbionts (found in *P. echinospica* and *L. barhami*) (Supplemental Table S5). The cis-pyruvate hydratase (*ACO*) that interconverts citrate to isocitrate via cis-aconitate was present in *Candidatus* Vondammii symbionts and in three QGON01 (*L. satsuma*) symbionts. While all symbionts possess oxidative decarboxylases (*por*), from pyruvate to acetyl coenzyme A, the SZUA-229 symbiont lacks 2-oxoacid oxidoreductase (*korAB*) and 2-ketoglutarate coenzyme A oxidoreductase (*korABCD*), which are involved in the rTCA cycle (Li et al., 2018).

Intriguingly, this study identified the key genes involved in the CAM (Crassulacean acid metabolism, dark) carbon metabolism pathway in the SZUA-229 symbiont, namely phosphoenolpyruvate (PEP) carboxylase (*ppc*) and malate dehydrogenase (*mdh*) (Supplemental Table S5). CAM-dark metabolism typically functions in plant photosynthesis by accumulating CO_2_ concentrations at night for use in daytime photosynthesis (Hartwell, 2005), and the released CO_2_ is subsequently recycled by Rubisco (Borland et al., 2009). We speculate that the SZUA-229 symbiont, which lacks key genes necessary for the rTCA cycle, relies on CAM metabolism to accumulate CO_2_ and subsequently fix it via the CBB cycle. This adaptation is vital for satisfying the nutritional needs of both the tubeworm host and the symbiont. It was also noted that, in regard to glycolysis, the *Sulfurovum* symbiont lacks pyruvate kinase (*PK*), which catalyzes the conversion of PEP to pyruvate. However, the *Sulfurovum* symbiont possesses PEP carboxykinase (*pckA*), which catalyzes the conversion of oxaloacetate to PEP and participates in the gluconeogenesis pathway. Sun et al. found that the endosymbionts of the deep-sea mussel *Gigantidas platifrons* possess *pckA* genes critical for gluconeogenesis, suggesting that the symbiotic bacteria are more efficient at utilizing and accumulating carbon and electrons from methane in the seep environment (Sun et al., 2023). We hypothesize that the *Sulfurovum* symbionts may also utilize carbon derived from sulfides in the environment. The CBB cycle is the primary carbon fixation pathway in siboglinid symbionts. Symbionts experiencing energy depletion, such as in low-sulfur environments, may switch to using the rTCA cycle (Hügler and Sievert, 2011). Frenulata primarily inhabit anoxic, reduced sediments that have relatively low sulfur content compared to hydrothermal vents and cold seeps (Hilário et al., 2011). However, the SZUA-229 symbionts of Frenulata lack key enzymes associated with the rTCA cycle (*korA*, *korB*). Studies have shown that multiple copies of rTCA-related genes can lead to higher CO_2_ fixation rates (Li et al., 2018), which may help explain the larger population sizes of Vestimentifera and *Sclerolinum* tubeworms compared to those of Frenulata [The Frenulata tubeworm was discovered in the deep-sea sediment, with only one individual of the species found, whereas Vestimentifera and *Sclerolinum* tubeworms are typically found in clusters with large populations (Li Y. et al., 2015)].

##### 3.3.2.2 Nitrogen metabolism

Although siboglinid symbionts cannot fix nitrogen, they can perform nitrate reduction, denitrification, and ammonia assimilation (Liu et al., 2020). Nitrate reduction is thought to be the main pathway through which tubeworms acquire nitrogen (Minic and Hervé, 2004). In all siboglinid symbiont genomes examined in this study, only the respiratory nitrate reductase (*napAB*) responsible for nitrate reduction was identified (Figure 5b), while cytochrome c nitrite reductase (*nirB*, *nirD*, *nrfA*, *nrfA*, and *nrfH*) involved in dissimilatory nitrate reduction was not detected (Supplementary Table S5). Additionally, respiratory membrane-bound nitrate reductase (*narGHI*) was identified in *Candidatus* Endoriftia and five QGON01 symbionts (found in *S. jonesi*, *L. luymesi*, and *L. anaximandri*). This enzyme can catalyze the reduction of nitrate to nitrite, along with its associated electron carriers. In *L. anaximandri*, only three symbionts collected from cold seeps had *narGHI*. Notably, *Candidatus* Vondammii symbionts were found to lack nitrite reductase (*nirS*), nitric oxide reductase (*norBC*), and nitrous oxide reductase (*nosZ*), enzymes involved in the denitrification process. Only the SZUA-229 symbiont contained a complete set of key genes (*nasC*, *nasD*, and *nasE*) required for nitrate assimilation. These results show that tubeworm symbionts likely rely on bioavailable forms of nitrogen (primarily nitrate) for growth and possibly use the same enzymes for assimilatory and denitrification processes. Previous studies have shown that nitrate from the environment can be acquired by the host tubeworm for use by the symbionts (Liao et al., 2014). The symbionts reduce nitrate to ammonium, which is utilized for the growth and biosynthesis of both the symbionts and the host (Girguis et al., 2000; Robidart et al., 2011). Nitrate is highly abundant in deep-sea hydrothermal vents and cold seeps environments (Lee and Childress, 1994). *Sclerolinum* tubeworms are primarily found near active seepage sites (Xu et al., 2022), suggesting their relatively low demand for reducing substances. As a result, the *Candidatus* Endoriftia and QGON01 symbionts exhibit greater enrichment of genes related to nitrogen metabolism (Supplementary Figure S9). Studies have proposed that, under conditions of low nitrate availability, the Nap system can compensate for the low efficiency of *NarGHI* enzymes, facilitating nitrate respiration (Gardebrecht et al., 2012). However, respiration via the Nap system typically generates less energy than the Nar system (Sparacino-Watkins et al., 2014), implying that *Candidatus* Endoriftia and some QGON01 symbionts likely produce more energy.

##### 3.3.2.3 Sulfur metabolism

Tubeworms thrive in environments rich in hydrogen sulfide (Tunnicliffe et al., 2003), which is toxic to tubeworms and inhibits the activity of cytochrome c oxidase (*COX*) in the mitochondrial electron transport chain, thereby affecting energy metabolism (Zusman et al., 2007). To detoxify hydrogen sulfide, tubeworms use hemoglobin to transport hydrogen sulfide to their symbionts in the trophosome. Prior research has shown that sulfide: quinone oxidoreductase (*SQR*) is the primary sulfur oxidation system (Reinartz et al., 1998). The findings of the present study confirm the presence of *SQR* in all siboglinid symbionts, with the enzyme relying on its prosthetic group, flavin adenine dinucleotide, to catalyze the oxidation of H_2_S to sulfur globules (S_0_) (Griesbeck et al., 2000). Sulfur oxidation in siboglinid symbionts depends on the reverse dissimilatory sulfite reductase system and the SOX system (Gardebrecht et al., 2012). The present study demonstrates that, except for the *Sulfurovum* symbiont, the sulfur metabolism pathways of all other siboglinid symbionts are highly conserved. Notably, although the *Sulfurovum* symbiont lacks many key genes related to sulfur metabolism, it contains serine O-acetyltransferase (*cysE*) and cysteine synthase (*cysK*), which are necessary for a complete cysteine synthesis pathway (Supplementary Table S5). Cysteine plays a crucial role in sulfide binding and protects cells from oxidative stress (Li et al., 2019). In the SOX system, all symbionts, except the *Sulfurovum* symbiont, possess key genes involved in thiosulfate oxidation, including *SoxYZ*, *SoxAX*, and *SoxB* (Figure 5b). Concurrently, the hemolybdenum protein *SoxCD* was only identified in the *Sulfurovum* symbiont (Supplementary Figure S10). The product catalyzed by *SoxCD* is hydrolyzed by *SoxB,* releasing a second sulfate molecule (Welte et al., 2009). However, the *Sulfurovum* symbiont lacks key genes, such as *SoxY*, *SoxAX*, and *SoxB*. Given that the *Sulfurovum* symbiont is the second symbiont type discovered in *L. satsuma*, these key genes are present in the QGON01 symbiont in *L. satsuma*. It is thus hypothesized that the metabolites between the two symbionts can be mutually utilized (Zimmermann et al., 2014). This exchange could result in an increase in sulfate content, thereby enhancing the availability of sulfur and promoting the sulfur metabolism pathway. Additionally, the symbiont *Candidatus* Vondammii is the only one found to possess a complete assimilative sulfate reduction pathway, which is suggested to result from the relatively low δ^34^S values of *Sclerolinum* tubeworms (Xu et al., 2022), implying that the assimilation of hydrogen sulfide by the symbionts may depend on sulfate-driven anaerobic methane oxidation (Feng et al., 2015). It is hypothesized that the symbionts of tube worms in hydrothermal environments primarily utilize hydrogen sulfide as the main energy source rather than thiosulfate (Markert et al., 2011). Therefore, the presence of the SOX system suggests that when sulfide concentrations are low, thiosulfate may serve as an alternative energy source for the tube worm symbionts. Additionally, siboglinid tubeworms can form intracellular sulfur globules for sulfur storage (Li et al., 2018). As a result, when hydrogen sulfide concentrations in the environment are low, the symbionts can maintain energy production by oxidizing intracellular sulfur compounds (Hand, 1987; Pflugfelder et al., 2005).

##### 3.3.2.4 Hydrogen oxidation

In this research on siboglinid symbionts, 22 genomes from the SZUA-229, *Candidatus* Endoriftia, and QGON01 genera were found to contain group 1 NiFe hydrogenases (*hyaA*, *hyaB*, and *hyaC*) (Figure 5b). These genes, which encode hydrogenases, enable the siboglinid symbiont to use hydrogen as a substrate for energy production (Petersen et al., 2011; Reveillaud et al., 2018). The present findings suggest that symbionts may utilize hydrogen as an alternative energy source (Kleiner et al., 2015). Interestingly, the results indicated that three genomes of the *Candidatus* Vondammii symbionts contained additional hydrogen oxidation pathways, specifically the NAD(P) (+)-reducing hydrogenases pathway. NAD(P)(+)-reducing hydrogenases comprise four subunits (Friedrich and Schwartz, 1993): *hoxH* and *hoxY* form the hydrogenase module, which catalyzes the oxidation/reduction of H₂ and facilitates electron transfer to and from the hydrogenase active site, while *hoxF* and *hoxU* constitute the diaphorase module, where electrons are ultimately captured and used to reduce NAD(P)(+) to generate NADH or NADPH. Moreover, *hoxF*, *hoxU*, *hoxH*, and *hoxY* share homology with *nuoF*, *nuoG*, *nuoD*, and *nuoB* of the NADH oxidative respiratory complex I, respectively (Zang et al., 2017). In the *Candidatus* Vondammii symbionts, this study identified *hoxF*, *hoxU*, and *hoxY* but not *hoxH* (Supplementary Table S5). While the absence of *hoxH* may slightly impact *hoxF* levels (Aubert-Jousset et al., 2011), the presence of the *hoxF* and *hoxU* complexes in the *Candidatus* Vondammii symbionts facilitates the H_2_ redox process.

Since the symbionts do not interact with the external environment, all their substrates and products are provided or eliminated by the host (Robidart et al., 2011). The three types of symbionts, *Candidatus* Endoriftia, QGON01, and SZUA-229, exhibit slight differences in energy metabolism, suggesting that regardless of the external environment, the hosts Vestimentifera and Frenulata provide a similar internal environment for the symbionts (Gardebrecht et al., 2012), thereby maintaining their normal function and growth. *Candidatus* Vondammii symbionts are highly sensitive to environmental changes and, influenced by the in-situ environment and the *Sclerolinum* host, the symbiont exhibits distinct sulfur oxidation and hydrogen oxidation strategies.

### 3.4 Symbiont nutrition

Siboglinid symbionts possess typical metabolic pathways for nutrient generation, including amino acid metabolism, cofactor/vitamin metabolism, and carbohydrate metabolism. These symbionts can produce 11 amino acids and 12 cofactors/vitamins (Supplementary Table S6).

Amino acids are essential for symbionts to synthesize nutrients and energy. Glycine not only supports growth but also plays a crucial role in various metabolic processes. However, high levels of glycine can lead to the production of toxic metabolites, such as aminoacetone and methylglyoxal (Kim et al., 2015). The glycine cleavage system regulates glycine catabolism, resulting in the production of CO_2_, NH_3_, NADH, and 5,10-methylene-THF (Kume et al., 1991; Nakai et al., 2005; Liu et al., 2021). In this study, key genes involved in the complete glycine cleavage system (*gcv* (*PA, PB,* and *T*) and *DLD*) were identified in 23 symbiont genomes, primarily in the SZUA-229, *Candidatus* Vondammii, *Candidatus* Endoriftia, and QGON01 genera (Supplementary Table S6). These four types of symbionts can utilize glycine as a carbon source for energy and biomass synthesis, as well as for detoxification.

Only the *Sulfurovum* symbiont, which contains the key genes involved in threonine biosynthesis (*lysC*, *asd*, *hom*, *thrB*, and *thrC*), can produce threonine, the crucial precursor required for isoleucine biosynthesis. Although the key genes involved in isoleucine biosynthesis (*ilvABCDEH*) are present in all symbionts, we hypothesize that these genes primarily facilitate isoleucine production through the valine/isoleucine biosynthesis (*ilvBCDEH*) pathway, rather than the isoleucine biosynthesis pathway. Only the *Sulfurovum* symbiont can use both pathways to synthesize isoleucine. Notably, *Candidatus* Vondammii symbionts can also convert pyruvate to 2-oxobutanoate to regenerate isoleucine via the isoleucine biosynthesis pathway (Supplementary Table S6). Additionally, the results reveal that siboglinid symbionts can synthesize lysine through two distinct pathways. First, *Candidatus* Vondammii and QGON01 symbionts synthesize lysine through the DAP aminotransferase pathway. Key genes involved in this pathway, including *lysAC*, *asd*, *dapABF*, and *LL-diaminopimelate aminotransferase*, were identified in 19 genomes. Alternatively, SZUA-229 and *Candidatus* Endoriftia symbionts synthesize lysine via the succinyl-DAP pathway, with key genes (*lysCA*, *asd*, *dapABDCEF*, and *argD*) identified in six genomes (Supplementary Table S6). This diversity aligns with the general strategy in which siboglinid symbionts utilize different pathways to synthesize the same biomolecule depending on environmental and physiological needs (details on the metabolism of other amino acids and cofactor/vitamin metabolism are provided in the Supplementary material). However, the symbionts encode few substrate-specific transporters, which results in an inability to effectively transport nutrients to the host (Yang et al., 2020). On the other hand, digestive enzymes are present in the host’s trophosome (Wippler et al., 2016), enabling it to obtain nutrients by digesting the symbionts. The ability of siboglinid symbionts to metabolize multiple amino acids and cofactors/vitamins reflects their capacity to provide nutrients to their hosts and sustain themselves autotrophically in extreme environments.

### 3.5 Homeostatic mechanisms in siboglinid symbionts

Despite the high oxygen buffering capacity of the tubeworm’s hemoglobin (Arp and Childress, 1983), elevated oxygen levels may still be transmitted to the symbiotic bacteria in the trophosome, potentially subjecting them to oxidative stress. We identified the alkyl hydroperoxide reductase *ahpC*, involved in oxidative stress response, in the symbionts of all 26 siboglinid species. Besides, siboglinid symbionts can supply reducing power through the oxidative branch of the pentose phosphate pathway, which helps maintain intracellular homeostasis via suppressing oxidative stress (TeSlaa et al., 2023). In this work, key genes (*G6PD*, *PGLS*, and *PGD*) involved in the oxidation stage of this pathway were detected in 21 symbiont genomes across the *Candidatus* Vondammii, *Candidatus* Endoriftia, and QGON01 genera. The *Sulfurovum* symbiont also possesses key genes (*rpe*, *rpiB*, *tktA*, *ALDO*, and *GPI*) involved in the non-oxidative stage of the pathway, suggesting that it can provide phosphorylated carbohydrates as precursors for ribose-5P synthesis (Miclet et al., 2001). Under nutrient-deficient conditions, siboglinid symbionts sustain growth through conserving energy. This study identified genes related to glycogen biosynthesis (*UGP2*, *glgC*, *glgA*, and *GBE1*) in 25 symbiont genomes in the *Sulfurovum*, *Candidatus* Vondammii, *Candidatus* Endoriftia, and QGON01 genera, reflecting their capacity to convert glucose 1P into glycogen for intracellular glucose homeostasis and a consistent energy supply (Moslemi et al., 2010).

Microbial terpenoids, primarily known as infochemicals, play a crucial role in intra- and inter-specific communication and interactions (Avalos et al., 2022). Infochemicals also aid microorganisms in adapting to oxidative, nitrosative, and nutrient stress (Avalos et al., 2022). Isoprene serves as the structural foundation for terpenoids (Bouvier et al., 2005). Among the 14 symbionts of QGON1, crucial genes related to C5 isoprenoid biosynthesis (*dxsr*, *ispDEFH*, *gcpE*, and *idi*) and C10–C20 isoprenoid biosynthesis (*idi*, *GGPS*, and *ispA*) were identified (Supplementary Tables S5, S6). Exposure to reactive oxygen species (ROS) induces oxidative stress in bacteria. Studies have shown that terpenoid compounds enhance the antioxidant potential of bacteria (Ostrovsky et al., 2003). Low temperatures reduce enzyme activity, and terpenes support bacterial growth under cold stress by modulating the fluidity of the cell membrane (Seel et al., 2018). Under nutrient limitation, some bacteria can utilize terpenoid compounds as a carbon source (Trutko et al., 2008). Therefore, we hypothesize that terpenoid compounds may enable the symbiont QGON01 to better adapt to the host’s internal environment and sustain its growth and reproduction (Bouvier et al., 2005).

This study identified genes encoding UDP-glucose (*glk*, *pgm*, *pmm-pgm*, and *UGP2*) and UDP-GlcNAc (*glk*, *GPI*, and *glmSM*, and *U*) in 21 genomes, primarily in the *Candidatus* Endoriftia and QGON01 genera. These symbionts inhabit the Vestimentifera tubeworm, whose trophosome is a highly vascularized organ (Hand, 1987; Uchida et al., 2023). UDP-glucose can differentiate into multiple blood cell lines (Kook et al., 2013), suggesting that the synthesized UDP-glucose may facilitate trophosome formation in Vestimentifera tubeworms, enabling rapid symbiont colonization within the tubeworms.

### 3.6 Secretory and prokaryotic defense systems

#### 3.6.1 Secretory systems

Siboglinid symbionts are thought to employ a Type II secretion system (T2SS) to evade phagocytosis and promote the bacterial invasion of host cells (Li et al., 2018). Hemolysin and chitinase, which are secreted via the T2SS, may aid bacteria in penetrating host cells and migrating toward newly formed cells, such as trophosome tissue (Li et al., 2018; Gao et al., 2023). The present research findings indicate that QGON01, *Candidatus* Endoriftia, and SZUA-229 possess essential genes involved in the T2SS system, while the *Candidatus* Vondammii symbiont lacks the complete gene set associated with T2SS (Supplementary Figure S11).

#### 3.6.2 Prokaryotic defense systems

**Figure 6:**
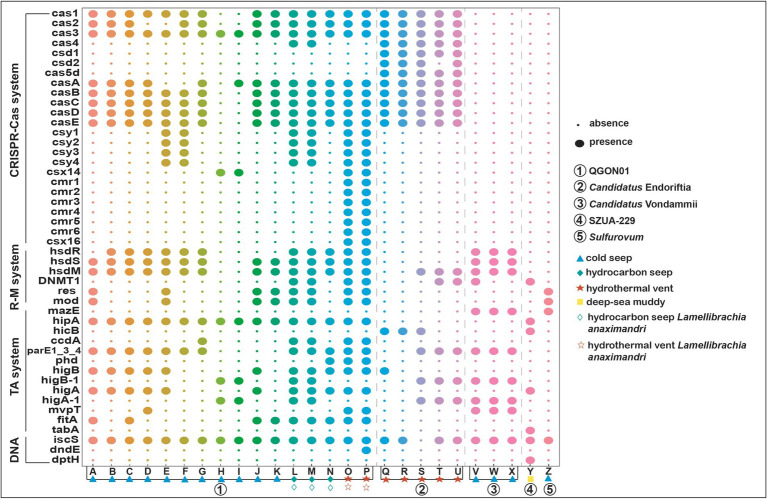
Gene distribution in siboglinid symbionts’ prokaryotic defense systems. This figure illustrates the presence of genes in the symbionts’ prokaryotic defense system. In the CRISPR-Cas system, *cas1* and *cas2* are Universal Cas proteins. In the Type I CRISPR-Cas system, *cas3* is a Type I signature Cas protein; *cas4* is a Subtype I-ABCD factor; *csd1*, *csd2*, and *cas5d* are Subtype I-C factors; *casABCDE* is a Subtype I-E factor; *csy1234* is a Subtype I-F factor; and *csx14* is a Subtype I-U factor. In the Type III CRISPR-Cas system, *cmr123456* belongs to the Subtype III-B factors and *csx16* is a Subtype III-U factor. The symbiont genomes corresponding to the alphabet letters are detailed in Supplementary Table S7 and the complete prokaryotic defense systems gene is displayed in Supplementary Figure S12. The blue triangle, green diamond, red pentagram, and yellow square represent habitat types. The circle colors are used for visual distinction and do not reflect specific classifications.

Siboglinid symbionts harbor numerous defense genes, with a primary focus on restriction modification systems, DNA interference, toxin–antitoxin systems, and CRISPR-Cas systems (Dupuis et al., 2013; Harms et al., 2018). Besides, Toll-like receptors are crucial components of the innate immune system in tubeworms, capable of recognizing invaders and activating immune responses (Sun et al., 2021). Meanwhile, the host controls the population size of symbionts through digestion, but during bacterial infections, the host’s immune system does not eliminate the symbionts (Ratzka et al., 2012). Instead, it protects the host from infection and may even participate in defending the symbionts from phage infections (Hinzke et al., 2019). The symbiont population is basically a bacterial monoculture within the tubeworm, and phage infection can be fatal. Our results indicate that only the symbionts of Vestimentifera tubeworms possess CRISPR-Cas-related genes, which may play a crucial role in protecting the symbionts from phage infections. The CRISPR-Cas system, comprising clustered regularly interspaced short palindromic repeats (CRISPR) and associated proteins (Cas) (Jansen et al., 2002; Westra et al., 2014). The present work demonstrates that the *Sulfurovum* symbiont lacks several genes associated with the prokaryotic defense system, a deficiency shared by the SZUA-229 and *Candidatus* Vondammii symbionts in relation to the CRISPR-Cas system (Figure 6). Interestingly, only the *Candidatus* Endoriftia symbionts were found to possess the *csd1*, *csd2*, and *cas5d* genes of the CRISPR-Cas system (I-C type; Makarova et al., 2011) (Figure 6). The presence of *Cas4* in *Candidatus* Endoriftia and QGON01 symbionts (found in *L. anaximandri* collected from cold seeps) enables these symbiotic bacteria to generate functional memory for future virus defense (Kieper et al., 2018). The QGON01 symbionts of the tubeworm *L. anaximandri* in hydrothermal vents contain a Type III-B CRISPR-Cas Complex (*cmr123456*) that is capable of RNA cleavage and RNA-activated DNA cleavage and thereby plays a pivotal role in adaptive immunity (Peng et al., 2015; Li et al., 2017b). Veesenmeyer reported that CRISPR-Cas proteins can facilitate colonization process of entomopathogenic nematodes Steinernema carpocapsae by the symbiotic bacterium *Xenorhabdus nematophila* (Veesenmeyer et al., 2014). Ponnudurai et al. identified the presence of CRISPR-Cas proteins in *Bathymodiolus azoricus* mussels that inhabit hydrothermal vents, indicating that these proteins may help symbiotic bacteria evade the host’s innate immune response during infection (Sampson and Weiss, 2013; Ponnudurai et al., 2017). Therefore, it can be assumed that siboglinid symbionts employ the prokaryotic defense system to defend against foreign phages and viruses, counteract extrachromosomal genetic materials, suppress gene expression, and promote their survival within the host (Patra et al., 2022; Bustamante-Brito et al., 2024).

However, this study lacks transcriptomic and proteomic data, and the limited number of genome sequences of *Sulfurovum* and SZUA-229 symbionts may introduce potential biases in the results. Additionally, there is no detailed discussion on the adaptive evolutionary effects of the host on the symbionts. Future research should focus on the interactions between animal hosts, their symbiotic bacteria, and the surrounding environmental microorganisms.

## 4 Conclusion

This study conducted a comparative analysis of siboglinid symbionts at the genus level to investigate functional heterogeneity and evolutionary relationships across various habitats and tubeworm taxa. The findings suggest that the genetic traits of tubeworm symbionts are primarily influenced by their habitats and hosts. The genomes of symbionts from different genera were found to exhibit significant differences, reflecting the influence of habitat and tubeworm species. Functional metabolic analyses highlighted key adaptations and nutrient sources for tubeworm symbionts in extreme environments. Siboglinid symbionts can adaptively utilize varying substrate conditions to evolve optimal energy acquisition mechanisms in different environments. Additionally, symbiont homeostatic mechanisms and the expression of genes related to prokaryotic defense systems play crucial roles in adaptive survival. Overall, the findings of this study offer insights into the adaptive evolutionary processes in siboglinid symbionts across different genera.

## Data Availability

The original contributions presented in the study are publicly available. This data can be found here: https://www.ncbi.nlm.nih.gov/, accession number: PRJNA1241460.

## Footnotes

^1^ https://github.com/labgem/PPanGGOLiN
