# Supplementary material for: Comparative metagenomic analysis reveals the adaptive evolutionary traits of siboglinid tubeworm symbionts
Source: Front Microbiol. 2025 Apr 17;16:1533506. doi: 10.3389/fmicb.2025.1533506 (PMC12045306; doi:10.3389/fmicb.2025.1533506)
Supplement: Supplementary file 2 [file Data_Sheet_1.pdf]

Supplementary Information for:

Comparative metagenomic analysis reveals the adaptive evolutionary traits of siboglinid tubeworm symbionts

1 Supplementary Materials and Methods

Pangenomes analysis

Pangenomes are categorized into three types (Gautreau et al., 2020): (1) persistent genomes, which include gene families found in nearly all genomes. These genes are essential for vital metabolic pathways and define the metabolic and biosynthetic capabilities of taxonomic groups. (2) Shell genomes, which consist of gene families present at moderate frequencies. These genes are often acquired through horizontal gene transfer and encode functions related to environmental adaptation, pathogenicity, virulence, or secondary metabolite synthesis. (3) Cloud genomes, which comprise gene families present at low frequencies. Genes in this category are typically acquired through horizontal gene transfer and include antibiotic resistance genes and plasmid genes.

2 Supplementary Results and Discussion

ANI analysis of symbionts

Host species influenced symbiont selection more than habitat. We analyzed 26 symbiont genomes for Average Nucleotide Identity (ANI). The results showed that the (*Ridgeia* (GCA_001443665 and GCA_001443675), *Tevnia* GCA_000224925, *Riftia* (GCA_023733635 and GCA_000224455)) tubeworm symbionts that were only living in the Pacific hydrothermal vent were similar to each other (Supplementary Fig. S1). In

cold seep, most symbionts of the same host are similar to each other (*Escarpia laminate* (GCA_003660235 and GCA_003660225), *Paraescarpia echinospica* (GCA_008642435 and Hai6C03), *Lamellibrachia satsuma* (GCA_003934985, GCA_003934975), *Sclerolinum* (GCA_022530855, HMS1_CP099567 and SCTW1H)). Similarly, geographically close but different host symbionts exhibit similarity to each other (*Seepiophila jonesi* GCA_003349935 and *Lamellibrachia luymesi* GCA_003349875, ANI=1.0). Symbiont similarity also exists between geographically distant locations and different tubeworm species (*Lamellibrachia barhami* GCA_011947365 and *Paraescarpia echinospica*, ANI>0.97).

Lamellibrachia anaximandri can thrive in both hydrothermal vent and cold seep. It exists in hydrothermal vent (GCA_016756855, GCA_016756895) are similar to the symbiont in the cold seep (GCA_016756955), ANI>0.98. The presence of *Lamellibrachia anaximandri* symbionts, which differ in location but live in cold seep, is similar (GCA_016756865 and GCA_ 016756835, ANI=0.999). There are also *Lamellibrachia anaximandri* symbionts from the same location that are not similar (GCA_016756955 and GCA_016756865), with an ANI value of 0.93. This suggest that the *Lamellibrachia anaximandri* host can harbors different species of symbionts (Hinzke et al., 2021).

Amino acid metabolism

In addition, the PRPS gene responsible for PRPP biosynthesis (from ribose 5P to PRPP) is found in all siboglinid symbionts. PRPP serves as a crucial intermediate in cell metabolism and is utilized in the production of purine and pyrimidine nucleotides, histidine and tryptophan amino acids, the cofactor NAD, and certain aminoglycoside antibiotics (Hove-Jensen et al., 2016).

Serine and threonine metabolism: The key genes involved in Serine biosynthesis (*serA*, *serC*, *serB*) were identified in four genomes belonging to the SZUA-229 and *Candidatus* Vondammii genera. Additionally, genes related to Threonine biosynthesis (*lysC*, *asd*, *hom*, *thrB*, *thrC*) were found in the *Sulfurovum* symbiont (Supplementary

Table S4).

Arginine and proline metabolism: Key genes for the synthesis of arginine and proline were identified in 24 genomes, primarily found in four genera: SZUA-229, *Candidatus* Vondammii, *Candidatus* Endoriftia, and QGON01 (Supplementary Table S4). The synthesis of arginine begins with the encoding of *argABJCDEJ* to produce ornithine, followed by the encoding of (*OTC*, *argG*, *argH*) to convert ornithine into arginine. Proline is synthesized from glutamic acid through pro*BAC*.

Histidine and tryptophan metabolism: Key genes for histidine synthesis (*his GZEIAHFBCD*, *histidinol-phosphatase*, *IMPL2*) were identified in 25 genomes, primarily found in four genera: *Sulfurovum*, *Candidatus* Vondammii, *Candidatus* Endoriftia, and QGON01 (Supplementary Table S4). Similarly, key genes for tryptophan synthesis were also detected in 25 genomes, mainly distributed across four genera: SZUA-229, *Candidatus* Vondammii, *Candidatus* Endoriftia, and QGON01 (Supplementary Table S4). The symbiont initially produces tyrosine via the Shikimate pathway (*aroF*, *aroBQEKAC*), and subsequently converts tyrosine into tryptophan through *trpEGDFCAB* gene codes.

Metabolism of cofactors and vitamins

Our results demonstrate that all siboglinid symbionts possess genes involved in Riboflavin biosynthesis (*ribBAHEF*), NAD biosynthesis (*nadBACD*, *NADSYN1*), Coenzyme A biosynthesis (*coaXBCE*, *coaD*), Molybdenum cofactor biosynthesis (*moaABC*, *MOCS2B*, *mogA*, *moeA*), and Heme Genes of biosynthesis (*EARS*, *hemALBCDENJH*, *CPOX*, *PPOX*) as detailed in Supplementary Table S4. The genes responsible for Pimeloyl-ACP biosynthesis (*bioCH*, *fabBFGZI*), Biotin biosynthesis (*bioFADB*), Lipoic acid biosynthesis (*lipB*, *lipA*), Siroheme biosynthesis (*hemALBD*, *cysG*), and Ubiquinone biosynthesis (*ubiCADXIGHE*, *COQ7*) were predominantly found in 25 symbionts across four genera: SZUA-229, *Candidatus* Vondammii, *Candidatus* Endorifia, and QGON01 (Supplementary Table S4).

Other important metabolic pathways

In the oxidation stage of the pentose phosphate pathway (from glucose 6P to ribulose 5P), key genes *(G6PD*, *PGLS*, *PGD*) were identified in 21 symbiont genomes across the genera *Candidatus* Vondammii, *Candidatus* Endoriftia, and QGON01. Furthermore, key genes (*rpe*, *rpiB*, *tktA*, *TALDO1*, *GPI*) involved in the non-oxidative stage of the pentose phosphate pathway (from fructose 6P to ribose 5P) were also found in the *Sulfurovum* symbiont.

The results of lipid metabolism revealed the presence of key genes (*CDS1*, *CHO1*, *psd*) involved in Phosphatidylethanolamine (PE) biosynthesis across 25 symbiont genomes belonging to 5 genera. Additionally, the significant gene *pmtA* related to Phosphatidylcholine (PC) biosynthesis was identified in the genomes of five symbionts within *Candidatus* Endoriftia. In siboglinid symbionts, PE and PC are vital components of cell membrane phospholipids (Klug and Daum, 2014; Friedman et al., 2018). Also, PC play a role in *Candidatus* Endoriftia symbiont-host interactions (Klüsener et al., 2009).

The key genes *kdsABCD* involved in CMP-KDO biosynthesis for lipopolysaccharide metabolism were found in all siboglinid symbionts. Additionally, the *Sulfurovum* symbiont was discovered to possess the key gene *gmhABCD* responsible for ADP-L-glycero-D-manno-heptose biosynthesis. Furthermore, the key gene *rfbABCD* related to dTDP-L-rhamnose biosynthesis was identified in 25 genomes of *Sulfurovum*, SZUA-229, *Candidatus* Endorriftia, and QGON01 symbionts. All of the above-mentioned genes can be found in Supplemental Table S3.

References

Friedman, J.R., Kannan, M., Toulmay, A., Jan, C.H., Weissman, J.S., Prinz, W.A., et al. (2018). Lipid Homeostasis Is Maintained by Dual Targeting of the Mitochondrial PE Biosynthesis Enzyme to the ER. *Developmental Cell* 44(2)**,** 261-270.e266. doi:10.1016/j.devcel.2017.11.023.

Gautreau, G., Bazin, A., Gachet, M., Planel, R., Burlot, L., Dubois, M., et al. (2020). PPanGGOLiN: Depicting microbial diversity via a partitioned pangenome graph. *PLOS Computational Biology* 16(3)**,** e1007732. doi:10.1371/journal.pcbi.1007732.

Hinzke, T., Kleiner, M., Meister, M., Schlüter, R., Hentschker, C., Pané-Farré, J., et al. (2021). Bacterial symbiont subpopulations have different roles in a deep-sea symbiosis. *eLife*, 10, e58371. doi:10.7554/eLife.58371

Hove-Jensen, B., Andersen Kasper, R., Kilstrup, M., Martinussen, J., Switzer Robert, L., and Willemoës,

M. (2016). Phosphoribosyl Diphosphate (PRPP): Biosynthesis, Enzymology, Utilization, and Metabolic Significance. *Microbiol. Mol. Biol. Rev.* 81(1), doi:10.1128/mmbr.00040-16.

Klug, L., and Daum, G. (2014). Yeast lipid metabolism at a glance. *FEMS Yeast Res.* 14(3), 369-388. doi:10.1111/1567-1364.12141.

Klüsener, S., Aktas, M., Thormann Kai, M., Wessel, M., and Narberhaus, F. (2009). Expression and Physiological Relevance of Agrobacterium tumefaciens Phosphatidylcholine Biosynthesis Genes. *J. Bacteriol.* 191(1), 365-374. doi:10.1128/jb.01183-08.

3 Supplementary Figures

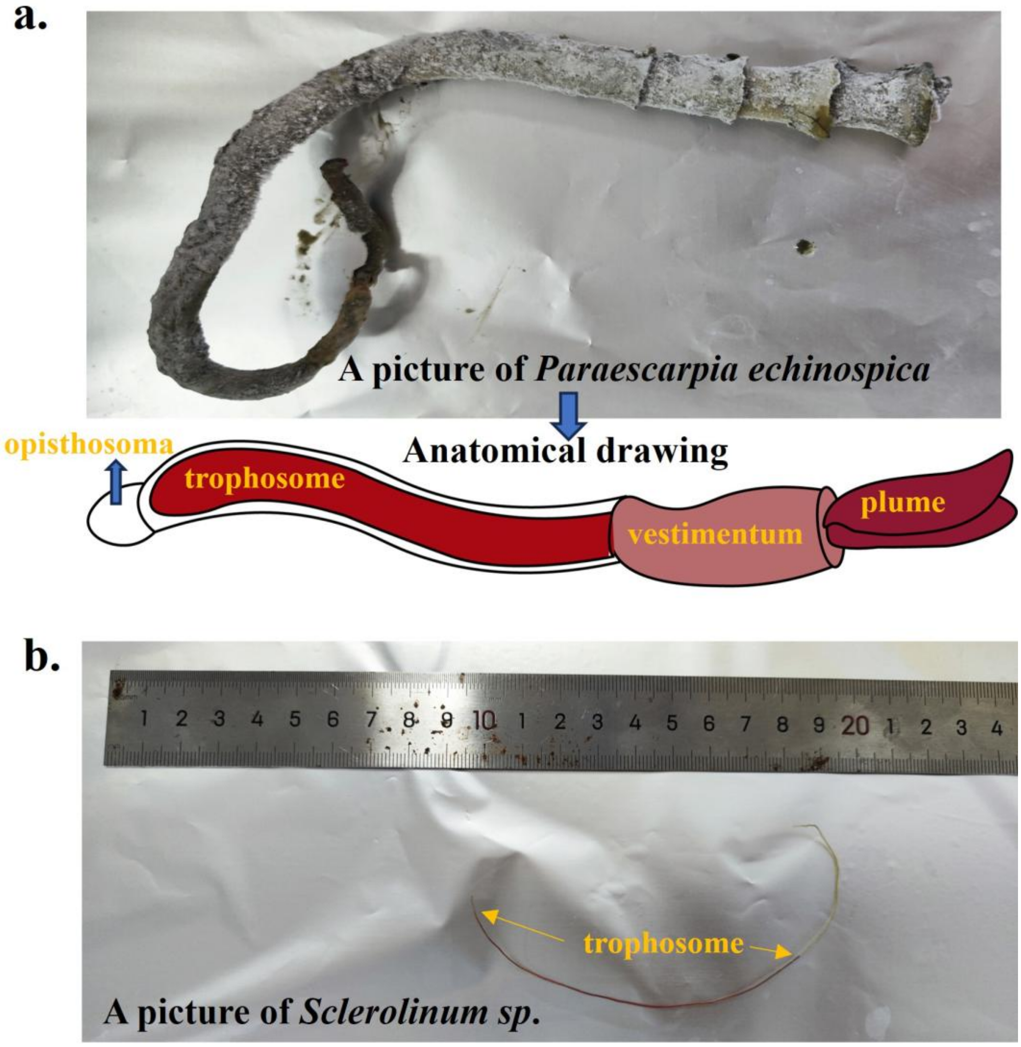

Supplementary Figure S1*:* a) The photo of *Paraeascarpia echinospica* tubeworm taken from the -80°C freezer and its anatomical diagram. b) The photo of *Sclerolinum sp.* tubeworm taken from the -80°C freezer.

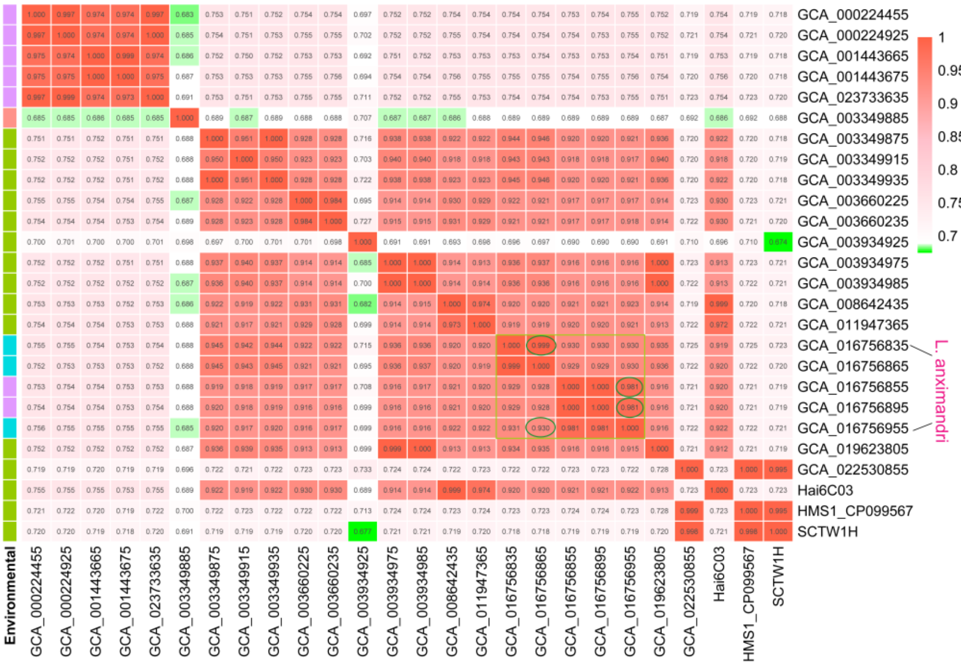

Supplementary Figure S2: The ANI similarity analysis of 26 siboglinid symbionts was calculated with the help of the ANIb model for all genomes two-by-two, and an ANI greater than 95% indicated that the symbionts were similar to each other.

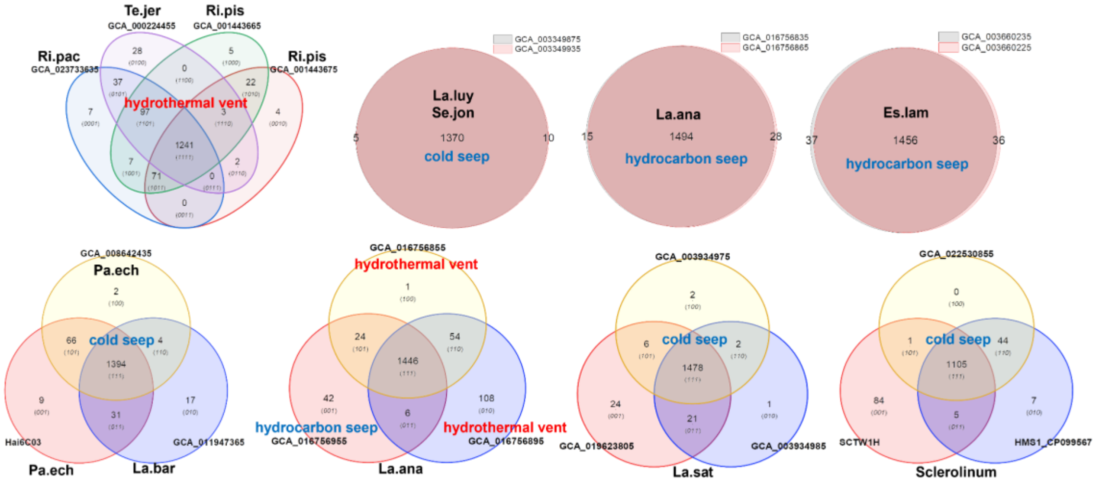

Supplementary Figure S3: Venn analysis of genetic differences between similar siboglinid symbionts.

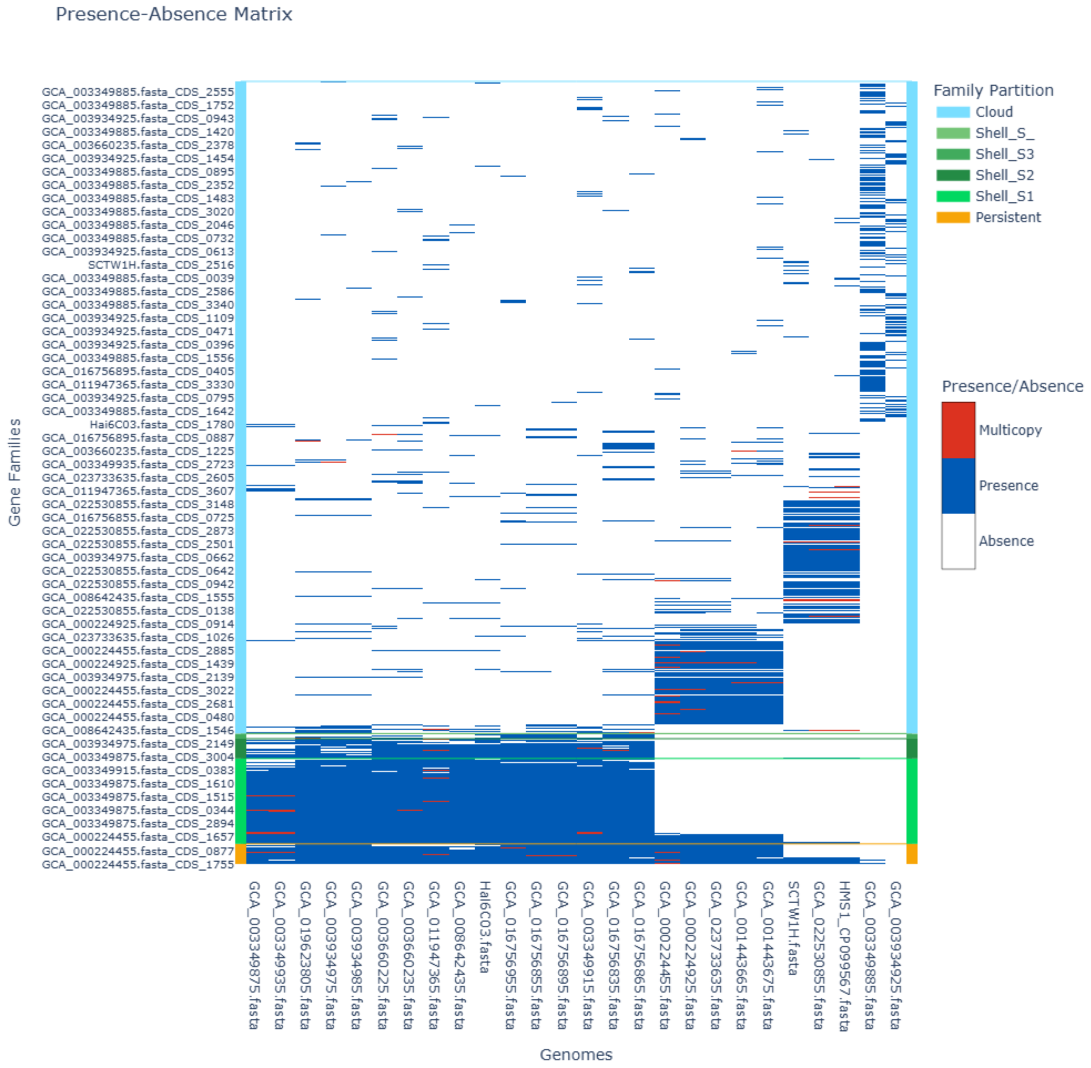

Supplementary Figure S4: The heatmap representing the gene families (y-axis) in the genomes (x-axis) making up your pangenome. Which illustrating the distribution of gene families within the persistent, shell, and cloud regions of siboglinid symbionts. The tiles on the graph will be colored if the gene family is present in a genome and uncolored if absent. The y-axis represents only a subset of gene families, rather than all gene families.

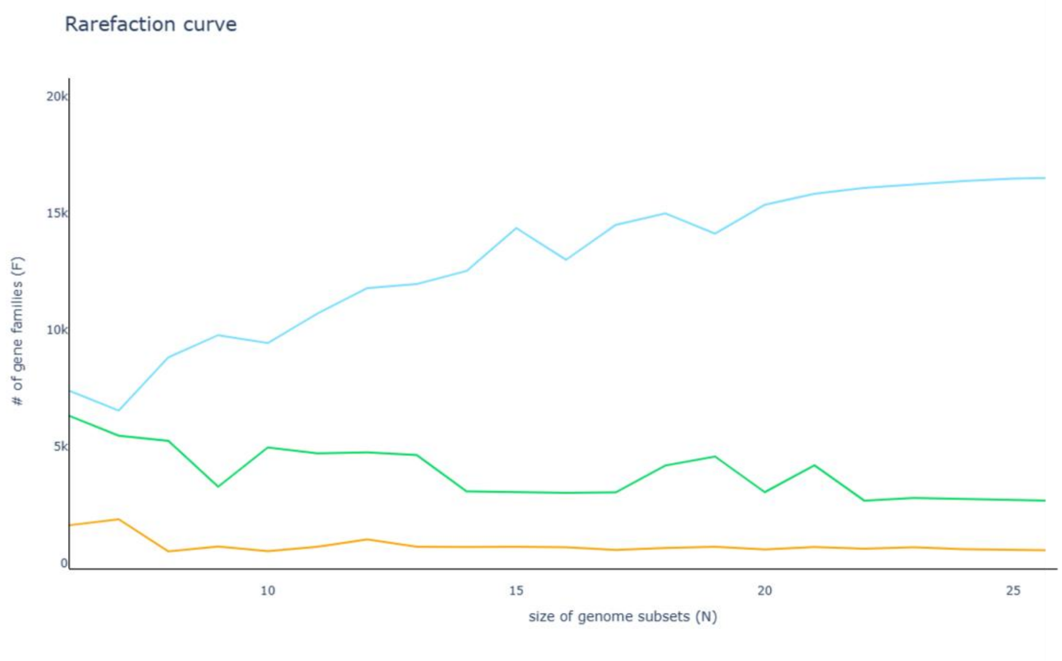

Supplementary Figure S5: rarefaction curve of the siboglinid symbionts, depicting the evolution of gene family counts per partition with the addition of more genomes to the metagenome. The blue line represents the 'cloud' region, the green line represents the 'shell' region, and the orange color represents the 'persistent' region.

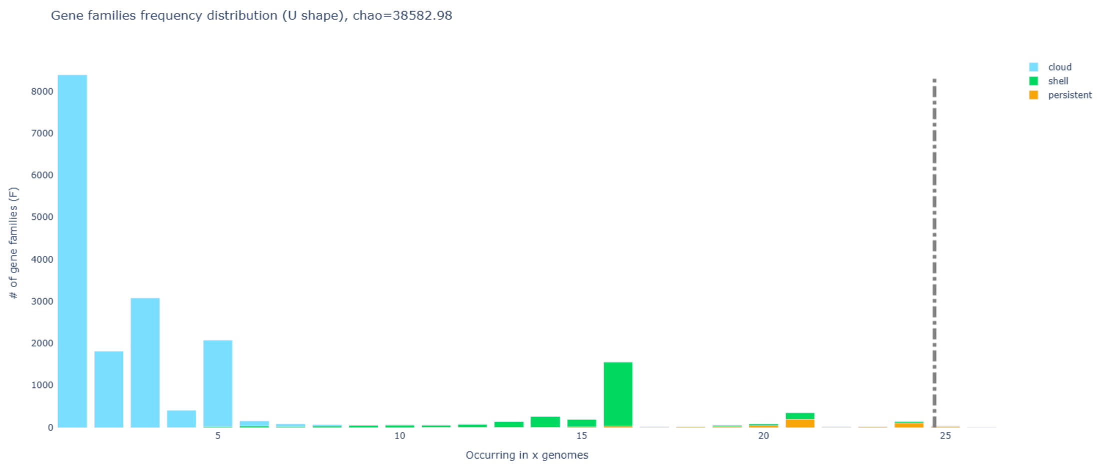

Supplementary Figure S6: U-shaped plot: gene families frequency distribution in pangenome. A U-shaped plot is a figure presenting the number of families (y-axis) per number of genomes (x-axis).

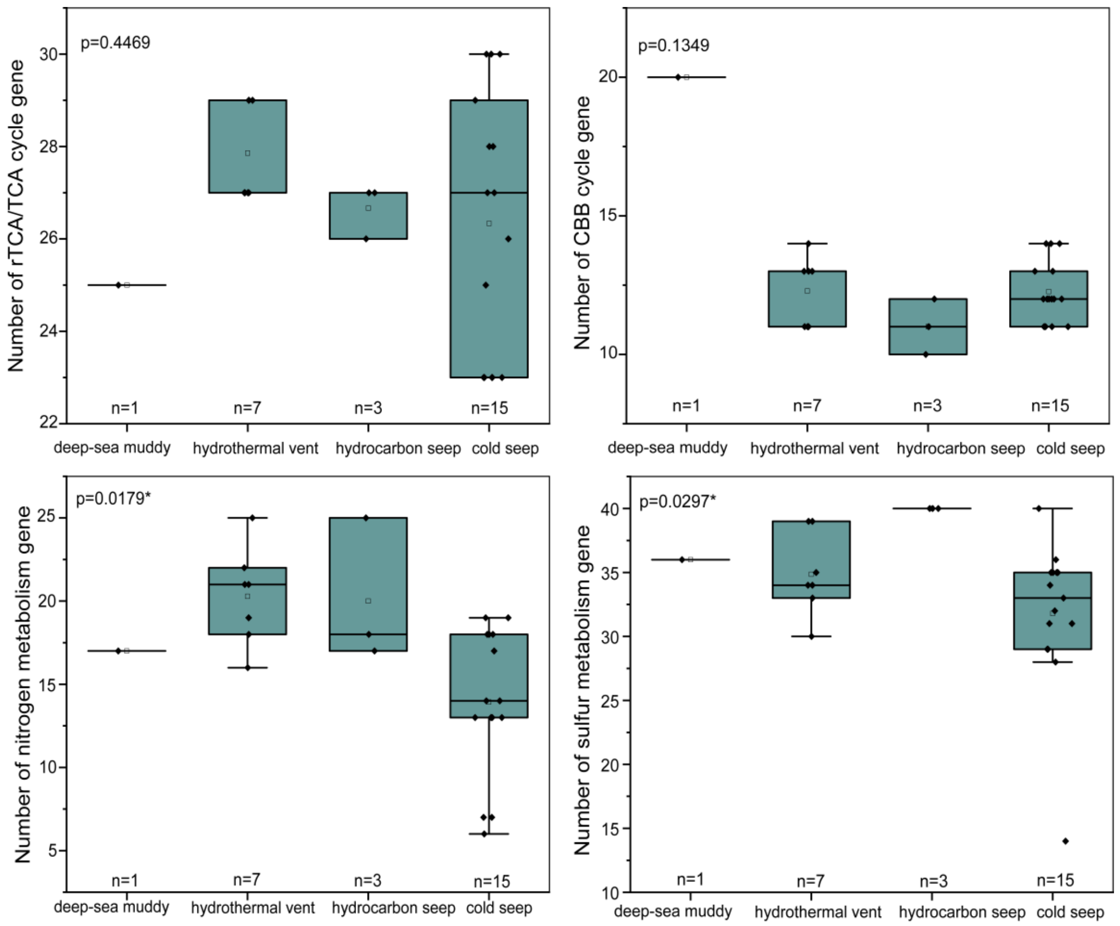

Supplementary Figure S7: Statistical analysis of the number of functional genes in symbionts across different habitats. The number of functional genes involved in sulfur metabolism and nitrogen metabolism shows significant differences. The analysis was conducted using the Kruskal-Wallis test statistic. p-values less than 0.05 or 0.01 indicate significant differences, with * denoting $p < 0.05$, and ** denoting $p < 0.01$.

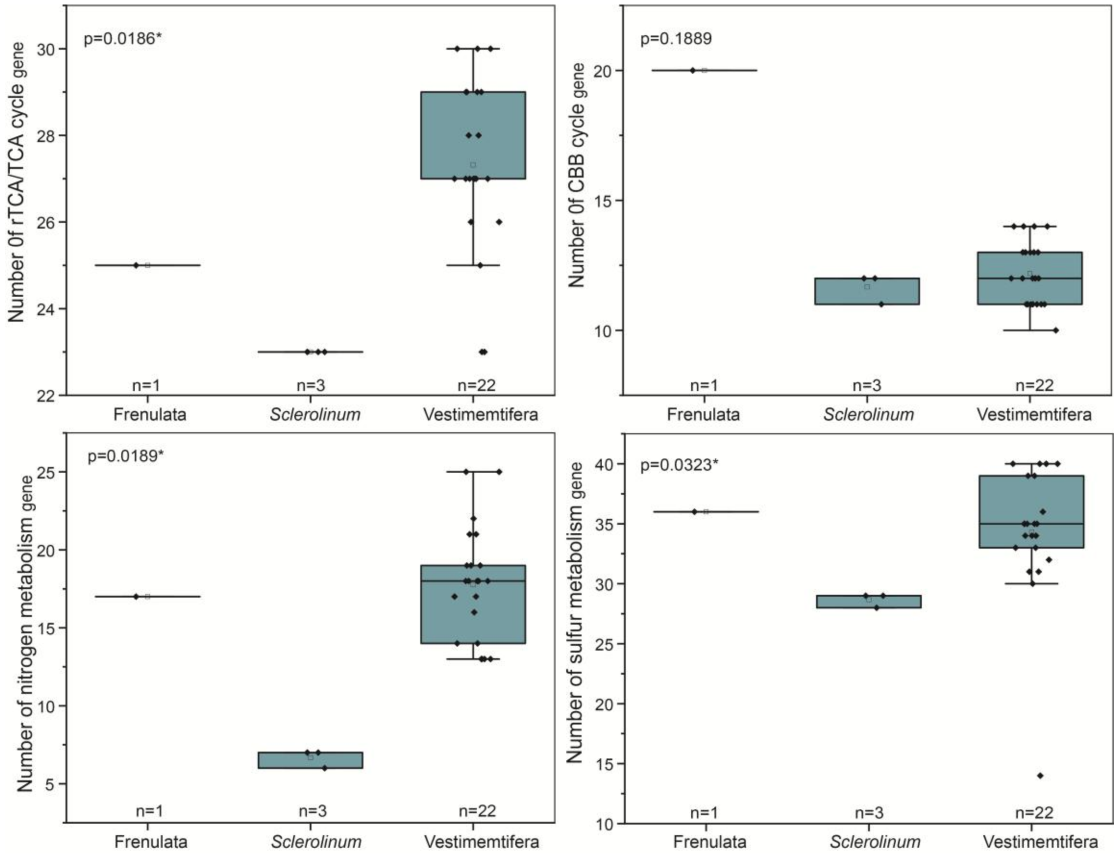

Supplementary Figure S8: Statistical analysis of the number of functional genes in symbionts from Vestimentifera, *Sclerolinum*, and Frenulata. The number of functional genes related to the rTCA/TCA cycle in carbon metabolism, as well as those involved in nitrogen and sulfur metabolism, shows significant differences. The analysis was conducted using the Kruskal-Wallis test statistic. p-values less than 0.05 or 0.01 indicate significant differences, with * denoting $p < 0.05$, and ** denoting $p < 0.01$.

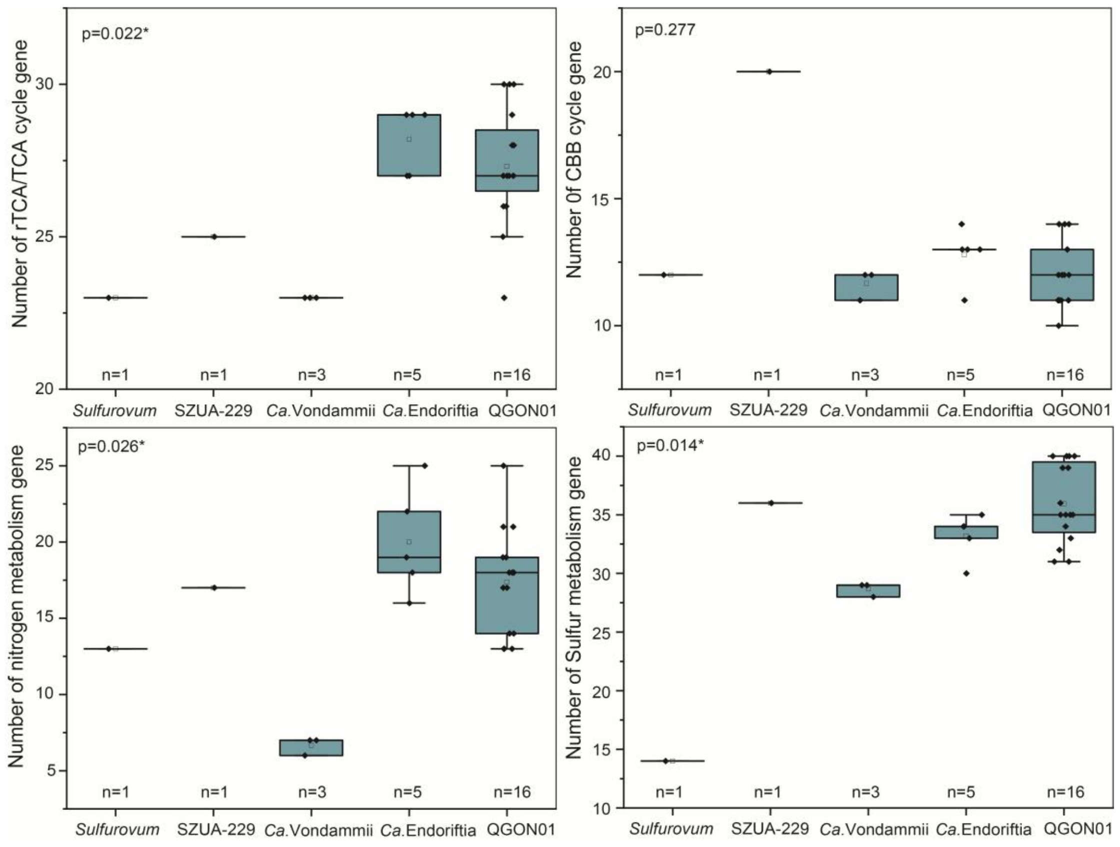

Supplementary Figure S9: Statistical analysis of the number of functional genes in symbionts from different genera. The number of functional genes related to the rTCA/TCA cycle in carbon metabolism, as well as those involved in nitrogen and sulfur metabolism, shows significant differences. The analysis was conducted using the Kruskal-Wallis test statistic. p-values less than 0.05 or 0.01 indicate significant differences, with * denoting $p < 0.05$, and ** denoting $p < 0.01$. *Candidatus* Vondammii (*Ca.* Vondammii), *Candidatus* Endoriftia (*Ca.* Endoriftia).

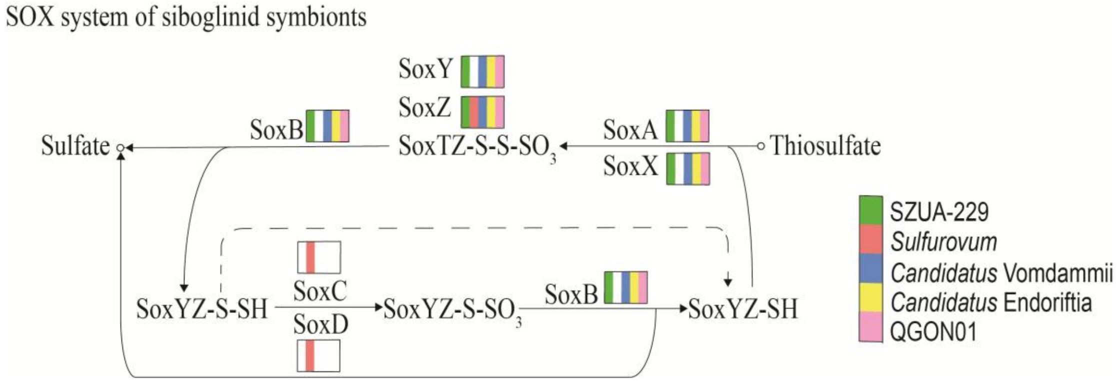

Supplementary Figure S10: Sox system of siboglinid symbionts. The boxes and colors

on the genes represent the presence or absence of the gene in the symbionts of the five genera.

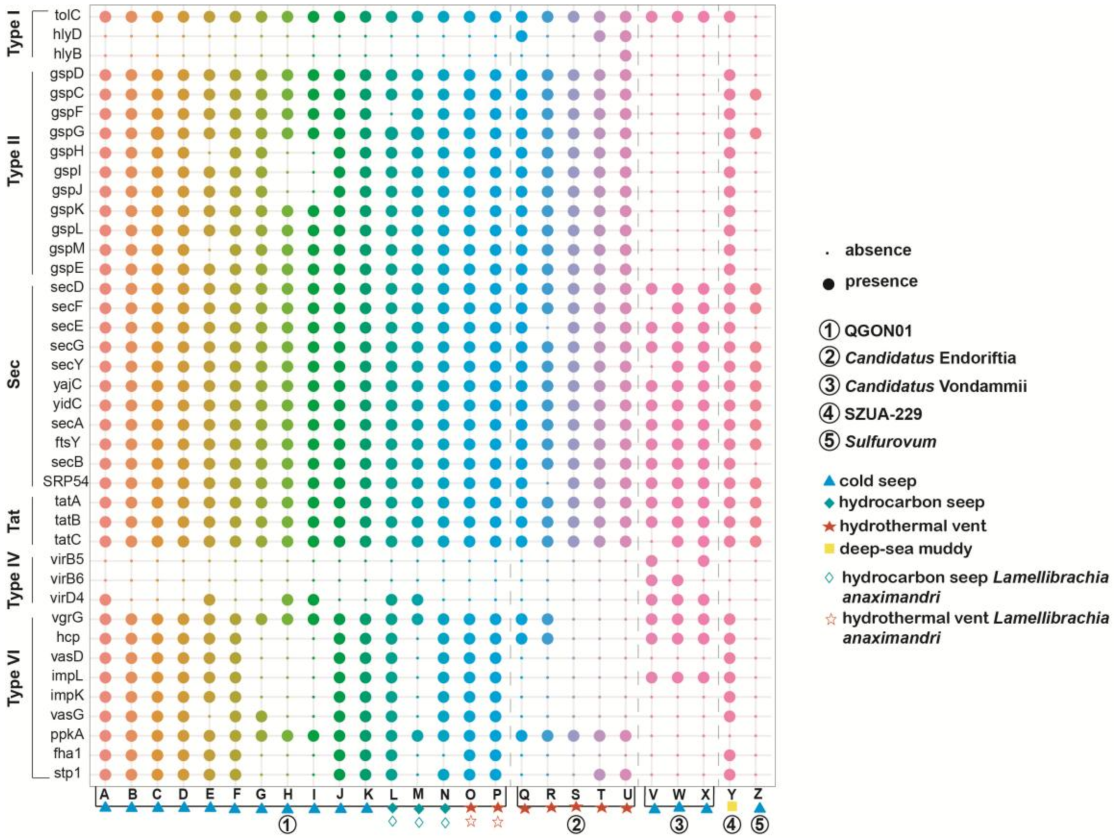

Supplementary Figure S11: Gene distribution in siboglinid symbionts' secretion systems. The circle colors are used for visual distinction and do not reflect specific classifications.

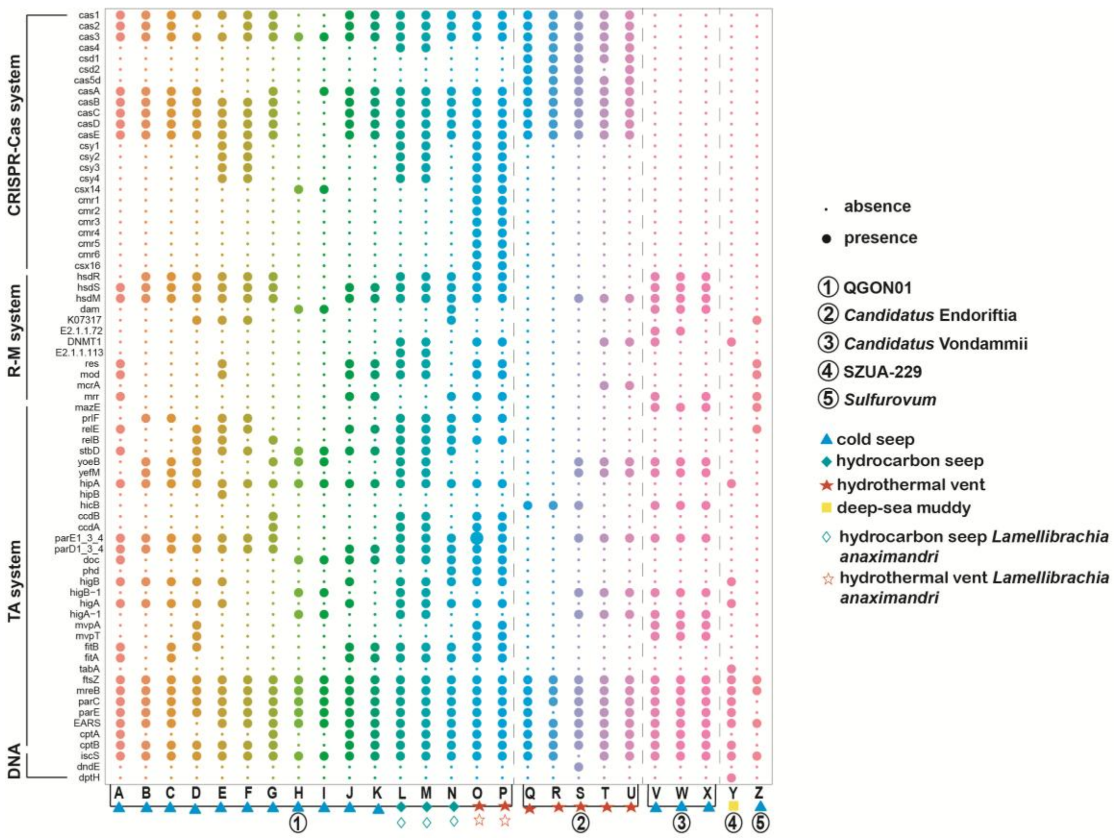

Supplementary Figure S12: Gene distribution in siboglinid symbionts' prokaryotic defense systems. The circle colors are used for visual distinction and do not reflect specific classifications.
